# Loss of E3 ligase *HvST1* function substantially increases distal crossover frequency

**DOI:** 10.1111/nph.70757

**Published:** 2025-11-30

**Authors:** Jamie Neil Orr, Sybille Ursula Mittmann, Luke Ramsay, Dominika Lewandowska, Abdellah Barakate, Malcolm Macaulay, Nicola McCallum, Robbie Waugh, Isabelle Colas

**Affiliations:** ^1^ Cell and Molecular Sciences The James Hutton Institute Invergowrie Dundee DD2 5DA UK; ^2^ Division of Plant Sciences University of Dundee at The James Hutton Institute Invergowrie Dundee DD2 5DA UK

**Keywords:** barley, crossover, meiosis, Sticky Telomere, synapsis, ubiquitin

## Abstract

- This study characterizes the function of a grass‐specific E3 ubiquitin ligase, *HvST1*, in regulating synapsis and crossover (CO) formation during meiosis in barley (*Hordeum vulgare*). In large‐genome cereals, COs are predominantly restricted to distal chromosomal regions, limiting genetic recombination and breeding flexibility. We aimed to identify genetic components regulating CO frequency and distribution.
- A frameshift mutation in *HvST1* was identified in the semi‐sterile barley near‐isogenic line BW233 through fine mapping of the *des12.w* locus. The causal role of this mutation was validated via CRISPR/Cas9 gene editing. HvST1 function was investigated using *in vitro* autoubiquitination and substrate ubiquitination assays, while meiotic progression was assessed using structured illumination microscopy and immunolocalization of key axis and synaptonemal complex (SC) proteins.
- Loss of *HvST1* function led to disrupted SC formation, persistent ASY1 signal, and aberrant ZYP1 polycomplex formation. Despite impaired synapsis, *Hvst1* mutants showed a significant increase in distal CO frequency. HvST1 was shown to ubiquitinate ASY1 *in vitro*, linking its activity to protein turnover at the chromosome axis.
- *HvST1* is critical for normal synapsis and CO regulation during meiosis in barley. Its loss disrupts SC progression but enhances distal CO formation, revealing a previously uncharacterized ubiquitination‐based mechanism modulating recombination in grasses.

## Introduction

Homologous recombination (HR) through resolution of double‐strand breaks (DSBs) as crossovers (COs) is an important trait for breeders, who rely on this mechanism of genetic exchange during meiosis to create novel crop varieties. In barley, COs occur mainly in distal chromosome regions leaving large sections of the genome almost devoid of meiotic recombination (Künzel & Waugh, 2002; Lambing & Heckmann, 2018). In plants, pairing of replicated homologous chromosomes in meiosis I is guided by the formation of DSBs catalysed by SPO11‐1 and SPO11‐2 (Grelon *et al*., 2001; Hartung *et al*., 2007). One strand of DNA at each side of the DSB is partially degraded (resected) by the MRE11‐Rad50‐NSB1 complex leaving 3′ single‐stranded DNA (Daoudal‐Cotterell *et al*., 2002; Wang *et al*., 2014). The RECA protein family, which includes RAD51 and DMC1 proteins, mediates strand invasion to create a ‘D‐loop’ (Pradillo *et al*., 2012; Da Ines *et al*., 2013). It is thought that D‐Loops in plants are resolved through double‐Holliday junction recombination intermediates to make COs (Neale & Keeney, 2006) via one of two pathways, resulting in either class I or class II COs. Class I CO resolution depends on the ZMM proteins (Zip1‐4, Msh4/Msh5, and Mer3; Higgins *et al*., 2005; Higgins *et al*., 2008b) and is subject to CO interference, a phenomenon that restricts the proximity of DSBs repaired as COs via this pathway (Capilla‐Pérez *et al*., 2021). Class II CO resolution is thought to depend on the MUS81 pathway (Higgins *et al*., 2008a) and is not subject to CO interference (Falque *et al*., 2009). In Arabidopsis and barley, it is estimated that only 5% of DSBs are resolved as COs (Higgins *et al*., 2012; Serra *et al*., 2018) and that 85% of these are resolved through the interference sensitive class I pathway. This introduces significant linkage drag which limits the ability of barley breeders to separate desirable and undesirable traits. Land races and wild relatives are a valuable resource to breeders for novel traits, such as disease or drought resistance. However, linkage drag can impose a severe yield or quality penalty when attempting to transfer these traits to elite varieties. Therefore, the ability to increase COs and break interference is appealing to plant breeders.

At the onset of meiosis in barley, the telomeres cluster to one side of the nucleus and the formation of the synaptonemal complex (SC, synapsis) is initiated (Higgins *et al*., 2012; Colas *et al*., 2017). The SC is a tri‐partite structure consisting of two lateral elements which form along replicated sister chromatids, helping to constrain them, and a central element which, beginning from the clustered telomeric region, forms between the lateral elements helping to physically link the paired homologous chromosomes during prophase I (Orr *et al*., 2021a). The SC is later dissolved before the first meiotic division, leaving the chromosomes held together by chiasma – the cytogenetic manifestation of COs – which help to ensure correct chromosome segregation during the second round of meiotic division (Orr *et al*., 2021a). Synapsis can be visualized using antibodies against key SC proteins, such as ASY1, a component of the lateral elements, and ZYP1, a component of the central element (Colas *et al*., 2017). Impairing the meiotic recombination pathway not only affects CO outcomes but can also affect synapsis, demonstrating the tight interplay of the two parallel mechanisms (Grey & de Massy, 2022). However, similar disruptions to these pathways can result in substantial differences in meiotic phenotype in different plant species. For example, barley desynaptic *des10*, which carries a mutation in MLH3 (Colas *et al*., 2016), displays abnormal synapsis contrary to the equivalent mutation in Arabidopsis (Jackson *et al*., 2006; Colas *et al*., 2016).

Recent studies have reported that mutations affecting the function or expression of *FANCM*, *RECQ4* and *HEI10* genes have the potential to increase COs (Mieulet *et al*., 2018; Serra *et al*., 2018; Arrieta *et al*., 2021), which is of great interest in large‐genome crops, such as barley, where the distal bias for COs is particularly pronounced (Künzel & Waugh, 2002; Lambing & Heckmann, 2018). To this end, we have exploited a collection of 15 *desynaptic* mutants that have been initially cytologically classified by their chromosome behaviour at Metaphase I in the early 1970s (Hernandes‐Soriano, 1973). Here, we show that one of these lines – BW233 – is mutated in the RING domain of a grass‐specific E3 ubiquitin ligase, that we call *HvST1* (*Sticky Telomeres 1*). We have shown that HvST1 is a functional E3 ubiquitin ligase and is capable of ubiquitinating ASY1 *in vitro*. Despite an abnormal synapsis progression, we found that loss of E3 ubiquitin ligase activity in *Hvst1* leads to an overall increase in COs which could be exploited during breeding programmes.

## Materials and Methods

### Plant material

Barley (*Hordeum vulgare*) BW233 is one of a collection of near‐isogenic lines (NILs) derived from 15 *desynaptic* mutants cytologically classified by their chromosome behaviour at Metaphase I in the early 1970s (Hernandes‐Soriano, 1973). These mutants have been backcrossed to a common barley cultivar Bowman to create NILs and initially genotyped to find the causal mutation for their meiotic phenotype (Lundqvist *et al*., 1997; Druka *et al*., 2010). Barley cv Bowman and NIL BW233 plants were grown under the following conditions: 16 h : 8 h, 18–20°C : 14–16°C, light : dark. For cytology, spikes of 1.4–2.5 cm were collected, and anthers were prepared as described previously (Colas *et al*., 2016). For fine mapping, 12 seeds from each of 16 families of the F_2_ population of BW233 × Barke were grown in 24‐pot trays. For the Kompetitive Allele Specific PCR (KASP) F_3_ recombination assay, 48 families of the harvested F_2_ BW233 × Barke population plants were chosen according to their genotypes across the *des12* interval. For the further 50K iSelect F_3_ recombination assay, eight seeds per F_2_ family were sown and grown as described above (24 families per genotype). DNA was extracted from young leaf tissue (two‐leaf stage) using the Qiagen DNeasy 96‐well plate Kit with the automated station QIAxtractor® or QIAcube®. RNA was extracted from 50 anthers in prophase I, using the Qiagen RNeasy Mini Kit. RNA quantity and quality were assessed with the NanoDrop 2000.

### Cytology

Metaphase spreads were performed as described by Higgins *et al*. (2012) with minor changes. Briefly, fixed anthers were incubated in 1% cellulase and 2% Pectolyase solution at 37°C for 30 min. The reaction was stopped by replacing the enzyme mix with cold (4°C) sterile distilled water (SDW). Nuclei preparation for immunocytology and Immuno‐fluorescence *in situ* hybridization (FISH) was performed as previously described (Colas *et al*., 2016).

#### Fluorescence *in situ* hybridization

Probes for centromeres (Bac7) and telomeres (HvT01) were amplified by PCR and labelled by nick translation with Alexa‐Fluor as described previously (Colas *et al*., 2016). Hybridization mix and denaturation were performed as described previously (Arrieta *et al*., 2021). Slides were washed in 2xSSC at room temperature (RT) for 10 min before digestion with 0.01% (w/v) pepsin in mildly acidified SDW for 45–90 s at 37°C. Slides were dehydrated using a series of ethanol concentrations (50, 70, and 90–100%) for 2 min each and left to air‐dry at RT. Hybridization mix (40 μl) was applied to the slides, and slides were incubated at 75°C for 4 min, then over night at 37°C in a dark humid chamber. Slides were washed and prepared as described previously (Colas *et al*., 2016).

#### Immunocytology

Slides were prepared as described previously (Colas *et al*., 2016, 2017, 2019). The primary antibody solution consisted of one or multiple antibodies as follows: anti‐HvASY1 (rabbit, 1 : 1000), anti‐HvZYP1 (rat, 1 : 500), anti‐HvDMC1 (rabbit, 1 : 500), anti‐HvMLH3 (rabbit, 1 : 500), anti‐HvHei10 (rabbit, 1 : 500), anti‐H3K27Me3 (no.: 07‐449; Merck Millipore Corp., Burlington, MA, USA), anti‐HvMLH1 (guinea pig, 1 : 200) diluted in blocking solution (1× PBS with 5% donkey/goat serum). For Immuno‐FISH, slides were washed in 2× SSC for 5 min after the secondary antibody incubation, followed by 10 min in 1× PBS. Slides were then fixed with 1% PFA for 10 min, rinsed briefly in 1× PBS before following the FISH protocol described earlier from the dehydration step.

#### Microscopy and imaging

Three‐dimensional structured illumination microscopy (3D‐SIM) and confocal images were acquired on a DeltaVision OMX Blaze (GE Healthcare) and LSM‐Zeiss 710, respectively, as described previously (Colas *et al*., 2016). Images were de‐convolved with the Imaris deconvolution module Clearview 9.5 and processed for light brightness/contrast adjustment with Imaris 9.5 (Randall *et al*., 2022).

### Positional gene identification

Initial genetic mapping used a custom 384 single‐nucleotide polymorphism (SNP) genotyping array using the Illumina BeadXpress platform on an F_2_ segregating population derived from a cross between BW233 (*des12.w*) and cv Morex, using the segregation of the semi‐sterile phenotype of *des12.w* as a Mendelian trait (Close *et al*., 2009; Druka *et al*., 2010). Nine hundred and thirty‐seven F_2_ plants from a BW233 x cv Barke cross were subsequently iteratively assayed using custom KASP® SNP assays (LGC Genomics GmbH) using the StepOnePlus™ real‐time PCR system (Supporting Information Fig. S3a, to be described later). Markers were initially designed using SNPs extracted from the manifest files of a 9k iSelect Genotyping platform (Comadran *et al*., 2012). Once all informative SNPs from the iSelect platform had been exhausted, comparative genomics with rice and Brachypodium were employed to identify candidate genes for further narrowing the interval. Polymorphisms in these genes were identified and scored on informative individuals through sequencing, using primers designed with Primer3 (Koressaar & Remm, 2007; Untergasser *et al*., 2012; Tables S1, S2). Gene sequences were retrieved from Ensembl Plants (Bolser *et al*., 2016) and aligned to barley genome assemblies (cv Morex, Bowman, Barke) using the IPK Barley BLAST Server (Colmsee *et al*., 2015; Deng *et al*., 2007). Contigs were aligned manually in BioEdit (Hall, 2001) to identify polymorphic regions. Primers were initially tested on cultivars Bowman, Barke, NILBW233, Betzes, Freja, Morex, and Klages and subsequently used on informative individuals from the BW233 × Barke F_2_ population.

### F_3_ meiotic recombination assay

The F_3_ meiotic recombination assay was based on KASP™ genotyping chemistry using 48 markers spanning three chromosomes (1H, 5H, and 6H; Table S3). The selected markers and frozen leaf punches were sent to LGC for KASP assay development. Five markers which consistently returned without a call for the genotype or were universally assigned to one genotype, were removed, leaving 43 markers. COs were counted at the junctions between loci with different allelic states along the length of each chromosome within regions that were heterozygous in the parental F_2_ using R. CO frequencies were calculated to generate linkage maps with MapChart (Voorrips, 2002). A subset of the F_3_ families was then analysed with the barley Illumina iSelect 50k SNP array (Bayer *et al*., 2017). In these data, monomorphic markers were removed, and a sliding window was used to correct for poorly mapped markers and resultant spurious double CO calls (markers with CO counts above five across all 95 plants were removed to account for this). One outlier semi‐sterile sample (AA/14/01/58/01:9A89) was removed having 63 observed COs. All codes used in 50K analysis and plotting are available online (see code availability statement).

### Gene model and expression

The *HvST1* gene model was constructed using the Barley Reference Transcriptome (BaRT) v.2.18 (Coulter *et al*., 2022). HvST1 meiotic expression was plotted using data from the Barley Anther and Meiocyte Transcriptome (BAnTr; Barakate *et al*., 2021).

### Gene cloning and expression

*HvST1*, truncated *Hvst1*, and *HvASY1* cDNA were synthesized with the SuperScript® III First‐Strand Synthesis System (Invitrogen) and amplified using nested PCR (Table S4) with High‐Fidelity Phusion or Q5 DNA polymerase (New England Biolabs, Ipswich, MA, USA). PCR products were ligated into pGEM®‐T Easy vector (Promega) and verified by Sanger sequencing using T7 and SP6 primers. Gateway cloning (Invitrogen) was used to transfer cDNA to pDEST‐HisMBP (Nallamsetty *et al*., 2009) for protein expression with the introduction of a TEV protease cleavage site allowing removal of HisMBP (Table S5).

### Protein expression and purification

Protein was expressed using *Escherichia coli* strain Rosetta™ 2(DE3) pLysS (Novagen, Now Merck KGaA, Darmstadt, Germany) grown to OD600 nm of *c*. 0.6. Cultures were cooled on ice before induction with 0.1 mM IPTG and grown overnight at 18°C and 220 rpm. Induced cell pellets were stored at −80°C until required and then lysed in BugBuster® Master Mix (Novagen) with cOmplete™ the EDTA‐free Protease Inhibitor Cocktail (Roche). The overexpressed protein was purified using HisPur NiNTA resin (Thermo Fisher, Waltham, MA, USA). For removal of the HisMBP tag, NiNTA elution buffer was exchanged with 50 mM Tris‐HCl, pH 8.0 using Pierce 10K MWCO spin columns (Thermo Fisher) before digestion with ProTEV Plus (Promega) overnight at 4°C with the addition of 1% Triton X‐100. Detergent was removed using a Pierce detergent removal spin column (Thermo Fisher); then, a second round of NiNTA capture removed the cleaved tag. Purified protein was exchanged into PBS (pH 7.4) using Slide‐A‐Lyzer G2 dialysis cassettes (Thermo Fisher) and stored at 4°C until use. The concentration of the purified protein was measured using the Pierce 660 nm microplate assay (Thermo Fisher) and absorbance read on the Varioskan Lux (Thermo Fisher).

### *In vitro* ubiquitination assays

Purified HvST1 interactions with E2 conjugating enzymes were determined by screening against a panel of human E2 conjugating enzymes using the E2‐scan plate (Ubiquigent). Reactions were evaluated for the accumulation of polyubiquitinated products via SDS‐PAGE followed by staining with SimplyBlue Safe Stain (Thermo Fisher) and western blotting with mouse anti‐ubiquitin conjugate antibody at a dilution of 1 : 10000 (Ubiquigent). Subsequent ubiquitination time‐course assays were conducted using histidine‐tagged UBE1, UBE2D4, bovine ubiquitin, and 8 mM ATP (Ubiquigent). HvST1 autoubiquitination time‐course assays and substrate (HvASY1) ubiquitination assays were incubated at 37°C for between 0 and 60 min. All control reactions were incubated at 37°C for 60 min.

These reactions were analysed via SDS‐PAGE followed by SimplyBlue Safe Stain (Thermo Fisher) and western blotting using mouse anti‐ubiquitin conjugate antibody as above, rat anti‐HvST1 antibody (1 : 200), mouse anti‐^6^His antibody (1 : 10000; Thermo Fisher), and rabbit anti‐HvASY1 antibody (1 : 5000; Agrisera; this study; AS21 4690). Excised gel fragments from autoubiquitination experiments were prepared as described previously (Lewandowska *et al*., 2019). Mass spectrometry was carried out by the Dundee University FingerPrints Proteomics Facility. The resulting raw files were processed and searched using MaxQuant (v.1.6.10.43; Tyanova *et al*., 2016), and the Andromeda peptide search engine (Cox & Mann, 2008; Cox *et al*., 2011) against the *E. coli* BL21 RefSeq proteome (GCF_014263375.1) combined with a custom set of sequences for the purified proteins added to the assay.

### TUBE capture

GST‐tagged tandem ubiquitin binding entities (GST‐TUBEs) were expressed and purified as described by Skelly *et al*. (2019). The HvASY1 ubiquitination assay was prepared as above and incubated for 2 h at 37°C. The reaction was passed through a 50 kDa size exclusion column (Thermo Fisher) to eliminate remaining free ubiquitin. GST‐TUBE was added to the column retentate to a final concentration of 200 μg ml^−1^ before overnight glutathione agarose (Thermo Fisher) capture at 4°C with gentle agitation. Each fraction of the glutathione agarose capture was retained and of equal volume. The captured protein was divided in two and half was incubated for 2 h at 37°C with broad‐spectrum deubiquitinating enzyme (DUB; LifeSensors, Malvern, PA, USA). The results of the assay were determined via SDS‐PAGE and western blotting as described above.

### Histone profiling

Bowman and BW233 anthers were collected by size at premeiosis, leptotene/zygotene, pachytene/diplotene as described previously (Barakate *et al*., 2021; Lewandowska *et al*, 2022). The nonmeiotic sample was prepared from collecting root tips from 5‐d‐old seedlings. Proteins were extracted as described previously, and histones were purified using Abcam Histone Extraction Kit – Rapid/Ultra‐pure (ab221031) as per manufacturer protocols followed by in trypsin in‐gel digestion (Lewandowska *et al*, 2022). Mass spectrometry was carried out by the Dundee University FingerPrints Proteomics Facility. The resulting raw files were processed and searched using MaxQuant (v.1.6.10.43; Tyanova *et al*., 2016), and the Andromeda peptide search engine (Cox & Mann, 2008; Cox *et a*l., 2011) against the *E. coli* BL21 RefSeq proteome (GCF_014263375.1) combined with a custom set of sequences for the purified proteins added to the assay.

### CRISPR/Cas9 construct

The *HvST1* coding sequence was searched using the online algorithm at http://www.broadinstitute.org/rnai/public/analysis‐tools/sgrna‐design. Two complementary oligonucleotides of 20 nucleotides were designed for each target with an additional four nucleotides at their 5'‐end for cloning into AarI restriction sites in the destination vector pBract214m‐HvCas9‐HSPT (Table S6). The complementary oligonucleotides were annealed using equal amounts of each oligo and ligated into the pBract214m‐HvCas9‐HSPT vector linearized with AarI. Inserts were verified by colony PCR and Sanger sequencing as described above. Plasmid integrity was checked by digestion with the restriction enzyme BglI (Thermo Fisher Scientific). The plasmid DNA was then transformed into *Agrobacterium tumefaciens* AGL1 strain containing the replication helper pSoup (http://www.bract.org/constructs.htm#barley) by electroporation. Transformed *Agrobacterium* clones from each CRISPR construct were used to transform barley cv Golden Promise immature embryos (Bartlett *et al*., 2008) in the plant transformation facility at The James Hutton Institute. Transgenic plants containing CRISPR constructs were regenerated under hygromycin selection.

### CRISPR/Cas9 screening and genotyping

T0 CRISPR plants were screened by PCR using primers flanking the target sites. Products were sequenced by Sanger Sequencing as described previously to verify mutations. Eight individuals were identified that had mutations at the expected gene location for the CRISPR01 construct. Twenty seeds of each were sown in 24‐pot trays soil in the glasshouse. At the two‐leaf stage, 2 mm leaf discs were collected, and plants were genotyped to identify plants that were homozygous for the identified mutations as described above. Additionally, plants were screened by PCR and gel electrophoresis for the presence or absence of the Cas9 transgene with Cas9‐F2 and Cas9‐R2 primers (Table S7). Five plants originating from three different T0 plants were identified to be homozygous and contain no Cas9 transgene. These lines were re‐potted in 9‐inch pots of soil to generate more seeds and tillers for cytology analysis. As a control, two plants that were found to contain no mutations were also included. Plants were grown for 6 wk until they reached meiosis and inflorescences were collected for chromosome spreads and immunolocalization as described above.

### Phylogenetic analysis

Proteomes from barley (*Hordeum vulgare*), rice (*Oriza sativa*), wheat (*Triticum aestivum*), maize (*Zea mays*), tomato (*Solanum lycopersicum*), cassava (*Manihot esculenta*), pineapple (*Ananas comosus*), soybean (*Glycine max*), clementine (*Citrus clementina*), Brassica oleracea, Tabaco (*Nicotiana tabacum*), *Arabidopsis thaliana*, *Amborella trichopoda*, *Physcomitrella patens*, yeast (*Saccharomyces cerevisiae*), *Caenorhabditis elegans*, *Drosophilla melanogaster*, zebrafish (*Danio rerio*), human (*Homo sapiens*), mouse (*Mus musculus*), and clawed frog (*Xenopus laevis*) were clustered into ortholog groups using Orthofinder (Emms & Kelly, 2019; v.2.3.3). HvST1 clustered only with proteins from included Poaceae proteomes. Further potential orthologs within Poaceae and the best possible alignments within *Arabidopsis* and monocot spp. were determined by BLAST alignment to the nonredundant archive (Altschul *et al*., 1990) and larger EggNOG (Huerta‐Cepas *et al*., 2019; v.5.0) precomputed orthologous groups. The longest isoforms of these proteins were aligned using Mafft (Katoh *et al*., 2002; v.7.266), and maximum likelihood phylogeny was constructed using IQ‐Tree (Minh *et al*., 2020; v.2.0.3) using model JTT + G4 with ultrafast bootstrapping (*n* = 1000). The resultant phylogeny was visualized using ggtree (Yu, 2020; v.2.5.1).

## Results

### The *des12.w* phenotype is due to a frame shift mutation in a novel E3 ubiquitin ligase

**Fig. 1:**
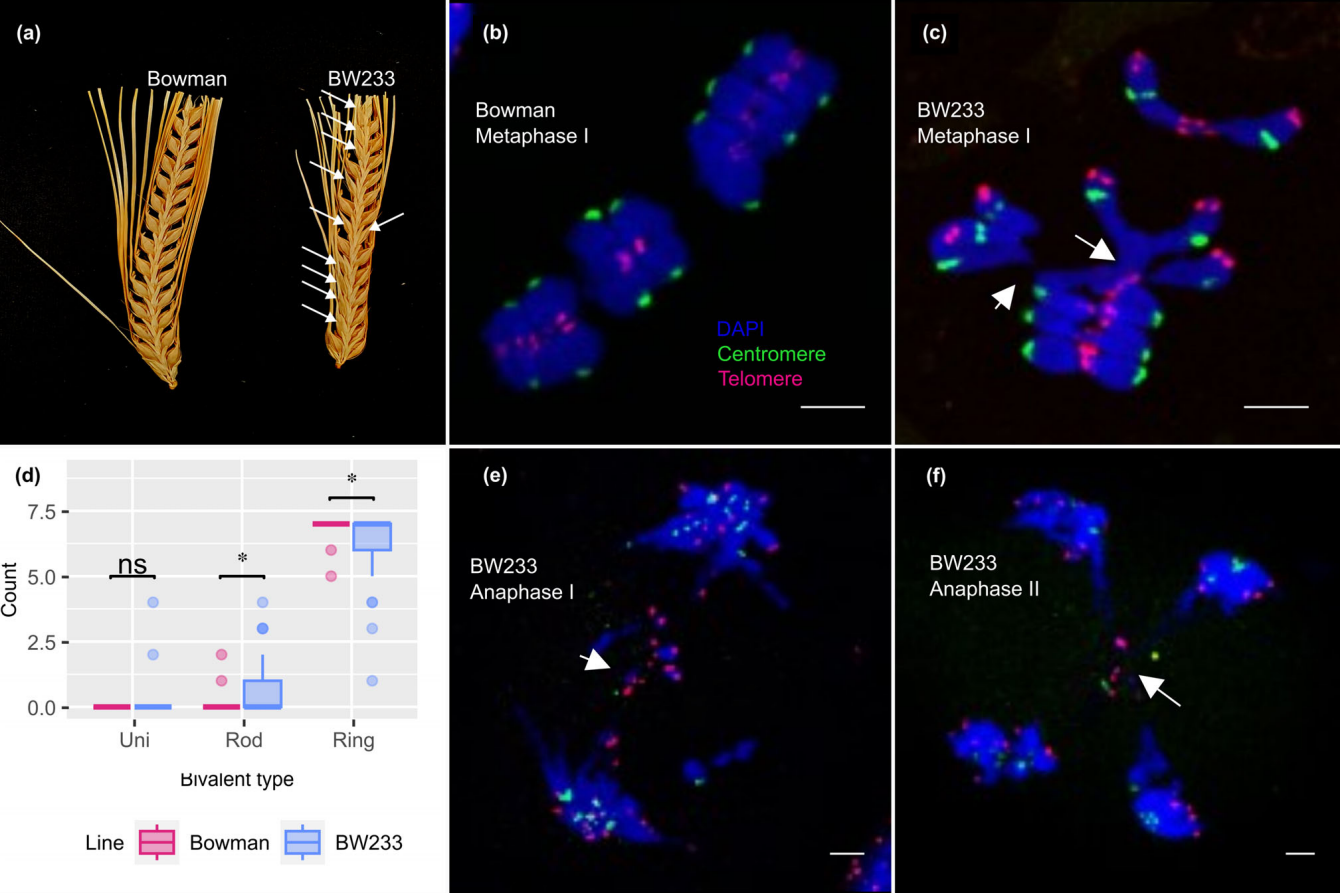
Reduced fertility and abnormal homologous chromosome pairing in Hordeum *vulgare* line BW233. (a) Spike fertility comparison between Bowman and BW233, showing missing seed indicated by white arrows in BW233. (b) Typical Bowman metaphase I fluorescence *in situ* hybridization (FISH) showing seven ring bivalents. (c) BW233 Metaphase I FISH, showing a mix of ring and rod bivalents, which are often interlocked at the telomere region (white arrows). (d) Box plot comparing the distribution of counts across univalent (Uni), rod bivalent (Rod), and ring bivalent (Ring) in Bowman and BW233 metaphase spreads with significance (*t*‐test) indicated above. It shows no significant difference (ns) for univalent, but a slight but significant increase in rod bivalents and a concurrent significant decrease in ring bivalents in BW233 compared with Bowman (indicated by ‘*’ above each category). The box represents the interquartile range (IQR), from the 25^th^ percentile (Q1) to the 75^th^ percentile (Q3), with the horizontal line marking the median. Whiskers extend to the minimum and maximum values within 1.5 × IQR, and dots beyond are potential outliers. (e) BW233 anaphase I FISH showing lagging chromosomes and chromosome bridges (white arrow). (f) BW233 anaphase II FISH showing interlocked chromosomes at the telomere region (white arrows). Chromatin (blue, DAPI), telomeres (red, HvT01 repeat), and centromeres (green, AGGGAG repeat). Bars, 5 μm; (ns: no significant).

BW233 (*des12.w*) is a semi‐sterile isogenic line of the barley cv Bowman (Fig. 1a) that carries a spontaneous mutation which originated in cv Freja. In contrast to Bowman (wild‐type (WT)) Metaphase I (Figs 1b, S1a, S2a), BW233 Metaphase I (Figs 1c, S1b, S2b) chromosomes are sticky and often interlocked in telomeric regions (arrows) and a statistically significant increase in mean rod‐bivalent and decrease in mean ring bivalent chromosomes (*t*‐test, Benjamini–Hochberg‐corrected *P* = 0.024 for both) is observed (Figs 1d, S2, S3). Chiasma counts from Bowman (*n* = 19) and BW233 (*n* = 44) meiocytes at Metaphase I showed a significant decrease in BW233 chiasma (Wilcoxon rank sum test, *P* = 0.0055) and altered distribution compared with Bowman (Fig. S3). Chromosome interlocks often persist at anaphase I (Figs 1e, S1b) and anaphase II in BW233 meiocytes (Figs 1f, S1b), potentially causing lagging chromosomes and mis‐segregation.

**Fig. 2:**
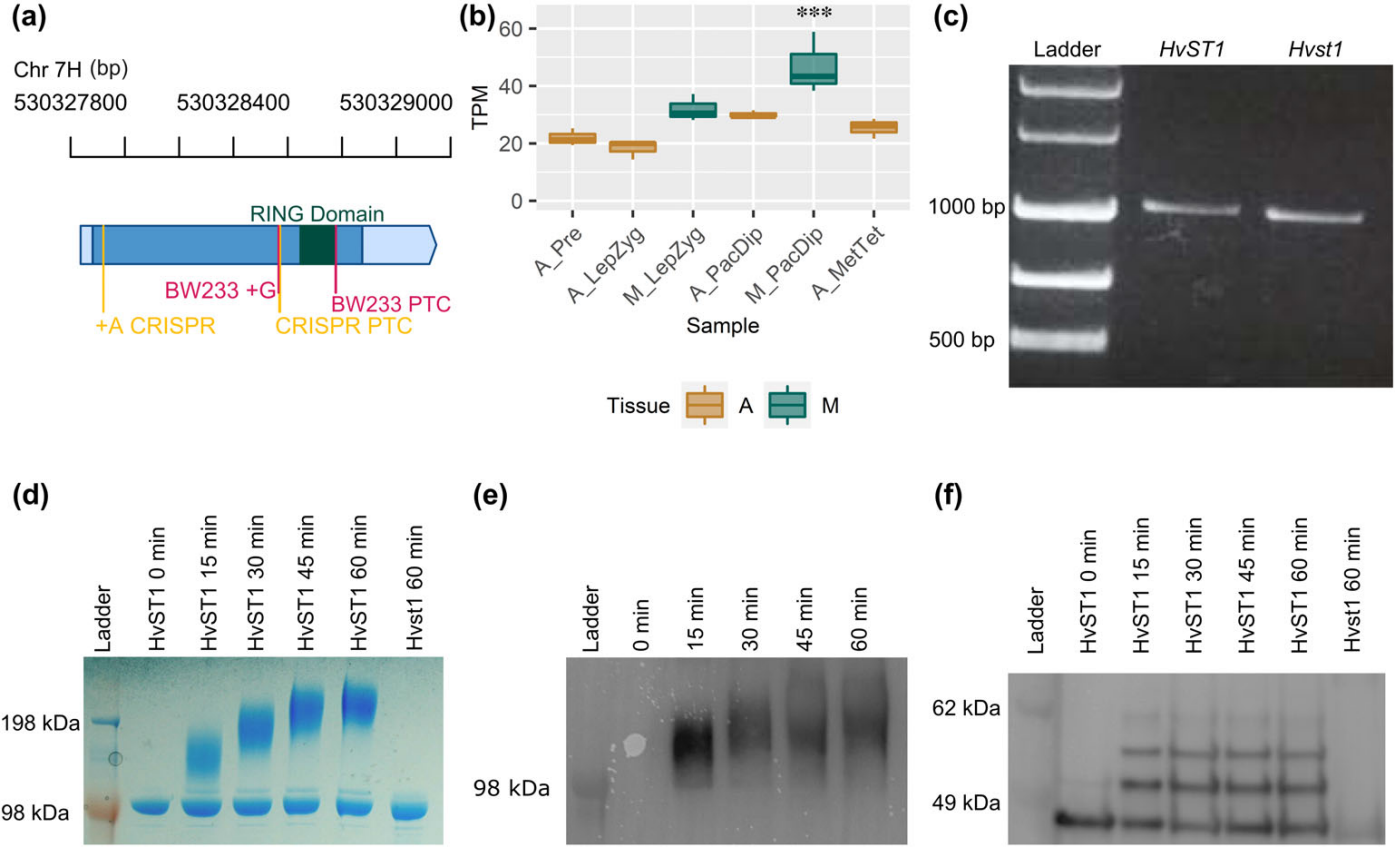
Hordeum *vulgare* HvST1 gene model, expression, and E3 ubiquitin ligase activity. (a) *HvST1* gene model derived from Barley Reference Transcriptome 2 (BaRT2v18; Coulter *et al*., 2022) RNA sequences mapped to the barley cv Barke genome with the BW233 (*Hvst1)* insertion (+G) and premature termination codon (PTC) indicated in dark pink and the CRISPR/Cas9 insertion and PTC indicated in yellow. The region encoding the RING domain is indicated in dark green. Wild‐type (WT) 3′ and 5′ untranslated regions (UTRs) are indicated in light blue while WT coding sequences are indicated in dark blue. The physical genomic coordinates (bp) are indicated above. (b) Expression profile of *HvST1* in time‐resolved anther and meiocyte expression data (Barakate *et al*., 2021) where A = Anther, M = Meiocyte, Pre = pre‐meiosis, LepZyg = Leptotene–Zygotene, PacDip = Pachytene–Diplotene, and MetTet = Metaphase–Tetrad. Each box represents the interquartile range (IQR) with the median indicated by a horizontal line, and whiskers extending to 1.5 × IQR. The meiocytes at Pachytene–Diplotene sample exhibit a significantly higher value compared with all other groups (**P < 0.001) as determined by ANOVA and Tukey's honest significant difference is indicated above the box. (c) Agarose gel of FL cDNA from RT‐PCR of *HvST1* from Bowman and *Hvst1* from BW233 anthers. (d–f) Autoubiquitination time‐course assays showing E3 ligase activity in HvST1 and loss of this activity in Hvst1 (d) Coomassie stained SDS‐PAGE showing accumulation of high‐molecular‐weight protein with HvST1 but not Hvst1 protein. (e) Anti‐ubiquitin conjugate probed western blot confirming the accumulated high molecular weight protein is polyubiquitinated. (f) Anti‐HvST1 probed western blot showing an increase in the mass of HvST1 in increments approximately the size of the ubiquitin monomer (8 KDa) but not of Hvst1.

To find the causal mutation of the *des12.w* phenotype, we used F_2_ populations of two crosses – BW233 x cv Barke and BW233 x cv Morex – scoring semi‐fertility as the segregating phenotype (Fig. 1a). We previously mapped the *des12.w* mutation to a 17 cM region on the long arm of chromosome 7H using segregating F2 plants in the BW233 x cv Morex population and a 384 SNP genotyping array (Druka *et al*., 2011). Fine mapping of *des12.w* was carried out using 937 F_2_ plants from the BW233 x cv Barke which were assayed through iterative KASP marker analysis to identify recombinants that segregated with the semi‐sterile phenotype (Figs S4a,b). Three hundred and eighty recombinants were identified within the 17 cM region previously identified of which 80 segregated with the proximal marker and 116 with the distal marker, confirming *des12.w* was within this interval and indicating that *des12.w* was closer to the proximal marker. Additional markers within this region were identified in a 9k iSelect genotyping platform (Bayer *et al*., 2017) and then, once these were exhausted, through resequencing of genic regions. These markers were used to iteratively refine the *des12.w* interval to a 0.5 cM region in which nine recombinants were identified, 7 and 2 segregating with semi‐sterility at the proximal and distal markers, respectively (Fig. S4b). This interval contained nine high‐confidence gene models (Fig. S4c) which were re‐sequenced to identify polymorphisms. This revealed a short exonic mononucleotide microsatellite in HORVU7Hr1g092570 (Morex v.3: HORVU.MOREX.r3.7HG0722700; Mascher *et al*., 2021) that contains an extra guanine in BW233 compared with WT cultivars Bowman, Barke, Freja, Morex, and Betzes, which induces a frameshift (Figs 2a, S5). HORVU.MOREX.r3.7HG0722700 is a 990‐bp single exon gene encoding a 330 amino acid RING finger domain containing putative E3 ubiquitin ligase that we have named *STICKY TELOMERES 1* (*HvST1*; Fig. S5). The frame shift in *des12.w* (*Hvst1*) introduces a premature termination codon in *HvST1* that eliminates the RING domain and compromises gene function (Figs 2a, S5). In our ‘barley anther and meiocyte transcriptome’ (Barakate *et al*., 2021) dataset, we found that *HvST1* expression was significantly enriched in staged meiocytes when compared to anthers, rising to peak expression in pachytene–diplotene (Fig. 2b). Sequencing Reverse Transcription Polymerase Chain Reaction (RT‐PCR) products from WT and BW233 (*Hvst1*) anthers revealed that *HvST1* and *Hvst1* produced mRNA encoding 330 and 298 amino acid proteins, respectively, confirming the introduction of a premature termination codon in the latter (Figs 2c, S5). Screening a further four independent semi‐sterile desynaptic mutants (Fig. S6) originating from different cultivars (Betzes, Klages) revealed that they also carried spontaneous mutations in the same microsatellite; BW240 (*des4.af*; from cv Klages), BW241 (*des4.a*, from cv Betzes) and BW229 (*des1.v*, from cv Freja) contain the same 1‐bp insertion, while BW242 (*des4.h*, from cv Betzes) has a 1‐bp deletion (Fig. S7). We generated CRISPR/Cas9 knockouts of this gene in *cv* Golden Promise which also caused semi‐sterility (Fig. S8).

### HvST1 is a grass‐specific functional E3 ubiquitin ligase

Clustering and phylogenetic analysis of HvST1 places it within a group of grass‐specific proteins (Fig. S9), that includes the rice orthologue CYTOSOL LOCALISED RING FINGER PROTEIN 1 (Park *et al*., 2019; OsCLR1; Os06g0633500). More recently, Ren *et al*. (2021) reported a study of DESYNAPSIS1 (DSNP1), an induced mutation by irradiation that introduced a 2‐bp deletion in the exonic region of LOC_Os06 g4270, causing a large reduction of COs which was already visible at metaphase and sterility. Alignment of OsCLR1 and DSNP1 revealed that they are identical. Within grasses HvST1/OsCLR1‐like proteins show strong C‐terminal conservation – including the RING domain – with a more variable N‐terminal region (Fig. S10). Outside the Poaceae alignment is limited almost entirely to the E2 interacting RING domain. *OsCLR1* is highly expressed under salt and drought stress (Park *et al*., 2019). However, this is not a trait shared by orthologues in sorghum, maize, and wheat (Park *et al*., 2019), nor is *HvST1* differentially expressed under these conditions (Fig. S11a–c), suggesting a unique gain of function in rice OsCLR1/DSNP1. While *HvST1* appears to be constitutively expressed in all tissues, expression is consistently higher in anther, spike, and microspore sample studies in the EoRNA database with the exception of relatively high expression in the third internode (Fig. S11d; Milne *et al*., 2021).

RING domain containing proteins comprise the largest group of E3 ubiquitin ligases, which confer substrate specificity to the ubiquitination cascade (Dove *et al*., 2016). The RING domain allows the E3 ligase to recruit E2 conjugating enzymes allowing the transfer of ubiquitin from the E2 to E3‐bound protein substrates (Dove *et al*., 2016; Iconomou & Saunders, 2016). Loss of this domain in *Hvst1* should therefore prevent interaction with E2 conjugating enzymes. In autoubiquitination time‐course assays, purified HvST1 interacted with the human E1 activating (UBE1) and E2 conjugating enzyme (UBE2D4), producing high molecular weight polyubiquitinated substrates visible via both Coomassie gel staining (Fig. 2d) and western blot with anti‐ubiquitin conjugate antibodies (Fig. 2e). Purified Hvst1 mutant protein did not interact with any E2 (Fig. 2d). Western blotting with anti‐HvST1 antibodies confirmed that HvST1 gained mass over the autoubiquitination time course (Fig. 2f). The identity of all protein bands in this assay was further confirmed by mass spectrometry (Fig. S12), confirming the specificity of the HvST1 antibody. Purified HvST1 protein interacted strongly with all human E2 conjugating enzymes in the UBE2D and UBE2E families (Fig. S13), and to a lesser extent with UBE2A, UBE2B, and UBE2N/V1. We conclude that HvST1 is a functional E3 ubiquitin ligase and that loss of the RING domain in *Hvst1* leads to loss of E3 ligase activity.

### *HvST1* mutants exhibit abnormal synapsis

**Fig. 3:**
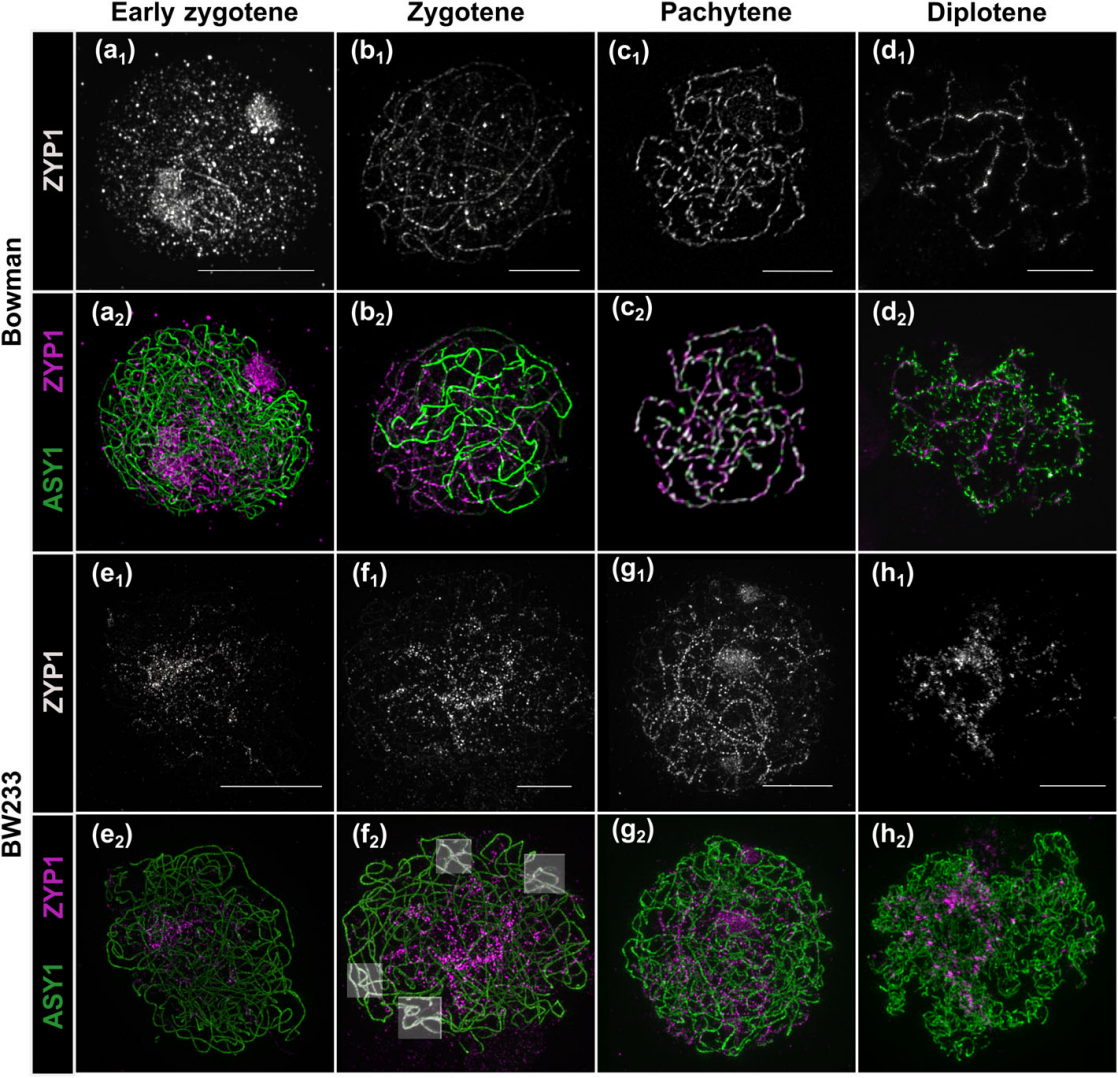
Synapsis in Hordeum *vulgare* lines Bowman and BW233. (a–d) Normal synapsis progression in Bowman. (a) ZYP1 polymerization starts at one side of the nucleus and elongates during (b) zygotene stage. Homologous chromosomes are fully aligned via ZYP1 at (c) pachytene and get separated at (d) diplotene exhibiting normal tinsel chromosomes. (e) Somewhat normal initiation of synapsis in BW233. (f) Zygotene cells of BW233 show abnormal elongation of the ZYP1 ‘cluster’ and unresolved interlocks (highlighted boxes). (g) BW233 pachytene‐like cell showing persistent HvZYP1 ‘clustering’. (h) BW233 diplotene cells exhibiting abnormal tinsel chromosomes. (a–h) ASY1: green, ZYP1: grey or magenta. Bars, 5 μm.

As *Hvst1* mutants are semi‐fertile, and display abnormal chromosome segregation (Fig. 1), we used SIM to compare synapsis progression between the WT and *Hvst1* mutants, using antibodies raised against axial (ASY1) and central element (ZYP1) proteins of the SC (Colas *et al*., 2017). Axis formation and the initiation of synapsis during early‐zygotene were comparable in WT (Fig. 3a) and *Hvst1* (Fig. 3e). By mid‐zygotene in the WT, most of the chromosomes are paired (Fig. 3b) and the typical tri‐partite structure of the SC is visible in the paired region (Phillips *et al*., 2012). By contrast, *Hvst1* meiocytes at the same stage exhibit faltering development with ZYP1 forming abnormal telomeric polycomplex‐like clusters (Fig. 3f). At pachytene stage, WT chromosomes are fully synapsed (Fig. 3c) but in *Hvst1* complete synapsis is compromised (Fig. 3g). During diplotene WT chromosomes display a normal tinsel configuration (Colas *et al*., 2017) as the SC is dissolved (Fig. 3d). However, in *Hvst1*, ASY1 unloading from the SC is perturbed and less organized with apparent misalignment and unpaired regions (Figs 3h, S14). The same synapsis defect was observed in insertion mutants BW229, BW240, and knockout Hv*st1* ^*CRISPR/Cas9*^, confirming cytologically that loss of E3 ligase activity in *Hvst1* causes the ZYP1 polycomplex‐like phenotype, independently from the original background (Figs S14, S15).

**Fig. 4:**
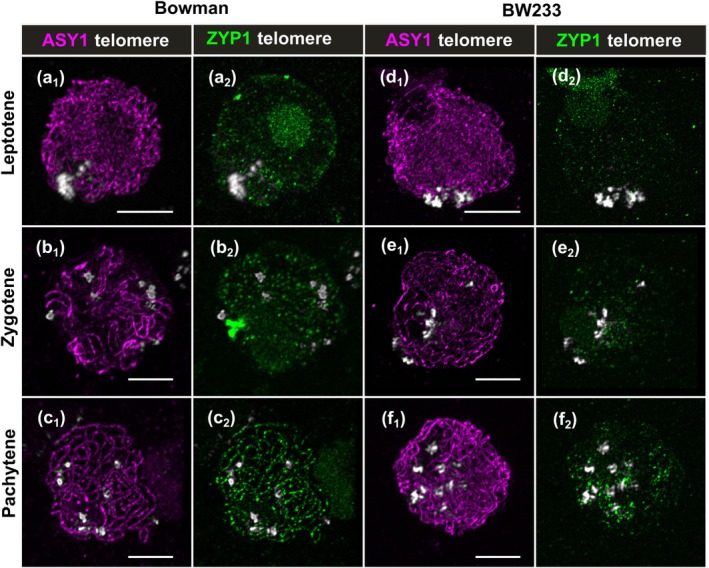
Telomere behaviour in Hordeum *vulgare* lines Bowman and BW233. At the leptotene stage, telomeres (grey) cluster at one side of the nucleus in both (a) Bowman and (d) BW233. As synapsis progresses from (b) zygotene to (c) pachytene stage, telomeres start to move around the nuclear periphery in Bowman. However, in BW233 (e) zygotene and (f) pachytene‐like stage cells, they are still located at one side of the nucleus. Bars, 5 μm.

We found that *Hvst1* mutants had a large number of chromosomal interlocks compared with the WT (Fig. 3f_2_, highlighted boxes), which could be the result of the synapsis delay or abnormal telomere organization. Therefore, we looked at telomere dynamics alongside synapsis and, in both genotypes, the telomeres cluster to one side of the nucleus (Fig. 4a,d), suggesting that telomere clustering is not compromised in the mutant. As synapsis progresses, the telomeres move around the nuclear envelope in the WT (Fig. 4b,c) but retain some polarization in *Hvst1* (Fig. 4e,f) indicating that compromised ZYP1 elongation and/or the presence of multiple interlocks affects their movement. The absence of HvST1 function does not therefore compromise initial chromosome alignment but does compromise both progression and dissolution of synapsis.

**Fig. 5:**
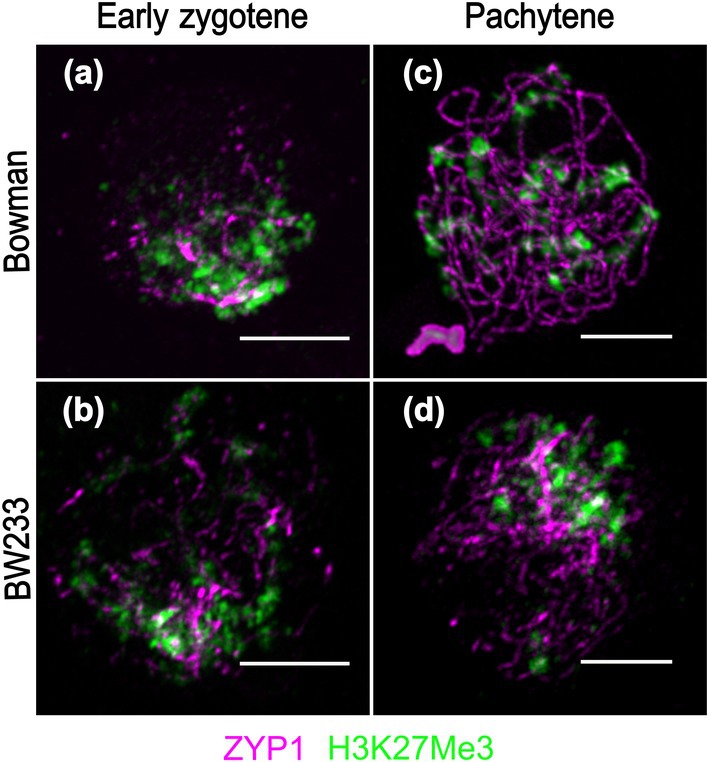
Chromatin behaviour and histone methylation in Hordeum *vulgare* lines Bowman and BW233. At the beginning of ZYP1 (magenta) polymerization, chromatin seems loose as shown by the diffuse H3K27me3 (green) around the telomere region in both (a) Bowman and (b) BW233. (c) In Bowman at pachytene, the H3K27me3 signal is orderly allowing identification of the telomeric regions. (d) In BW233, the H3K27me3 signal is more diffuse as a result of abnormal synapsis. Bars, 5 μm.

We followed synapsis progression and telomere movement using antibodies against ZYP1 and H3K27me3, respectively (Fig. 5), which is enriched in the telomere‐proximal region in barley (Baker *et al*., 2015). At early zygotene, ZYP1 starts to polymerize in both WT and BW233 and H3K37me3 signals are diffuse (Fig. 5a,b). At pachytene, H3K27me3 forms a clear pattern in the WT as described in Baker *et al*. (2015; Fig. 5e), but not in BW233 where H3K27me3 (Fig. 5d) suggests a potential change in chromatin state in the mutant. To confirm this, we profiled histone methylation in anthers at premeiosis, leptotene/zygotene, pachytene/diplotene and a nonmeiotic sample (root) by mass spectrometry. We identified histones H1, H2.A, H2.B, H3, H3 centromeric, and H4 in all samples as well as methylation in H2A, H2B, H3, and H4 (Fig. S16; Table S8). No significant quantitative difference in histone methylation (Kruskal–Wallis test, Benjamini–Hochberg correction) was observed (Fig. S16) between WT Bowman and BW233.

### COs are increased in *Hvst1* mutant progeny

**Fig. 6:**
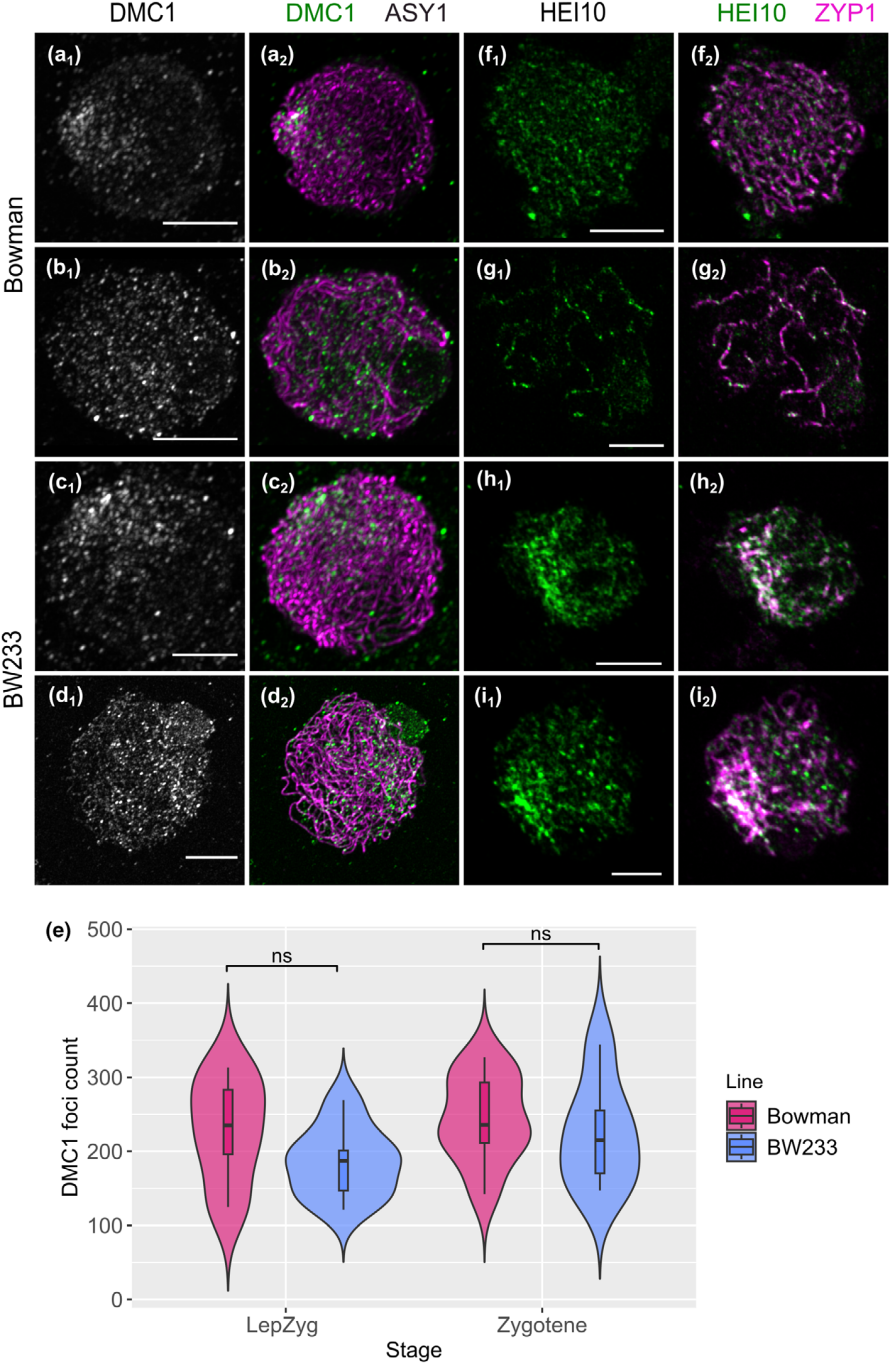
DMC1 and HEI10 behaviour in Hordeum *vulgare* lines Bowman and BW233. At the leptotene stage, DMC1 foci (green, grey) cluster at one side of the nucleus in (a) Bowman and disperse along the ASY1 axes (magenta) during (b) zygotene. In BW233, we see the same behaviour at (c) leptotene and (d) zygotene. (e) Violin plot of DMC1 foci count showing no significant difference between Bowman (magenta) and BW233 (blue). Internal boxplots indicate the median and interquartile range. Statistical comparisons between lines at each stage showed no significant differences (ns) Student's *t*‐test, *P* = 0.31 and 0.58. HEI10 foci load on ZYP1 axes in both (f, g) Bowman and (h, i) BW233, but they resolve as large foci in (g) Bowman pachytene cells while they cluster around the ZYP1 polycomplex in (h, i) BW233. Bars, 5 μm.

To understand the effect of *Hvst1* on COs, we first used antibodies raised against early and late meiotic HR markers. Co‐immunolocalization of HvDMC1 (early HR) and HvASY1 (chromosome axes) in both WT and mutant (Colas *et al*., 2019) showed that HvDMC1 behaviour was similar in both genotypes at the beginning of synapsis (Fig. 6a,e) but that HvDMC1 foci persist in the mutant compared with the WT (Fig. 6e) at later stages. However, DMC1 foci counts did not show a significant difference at the leptotene–zygotene transition or at zygotene (Fig. 6e; Student's *t‐*test, *P* = 0.31 and 0.58), suggesting that HvST1 is not involved in the recruitment of HvDMC1. However, the persistence of foci at later stages is indicative of the delay in synapsis. Co‐immunolocalization of MLH3 (late HR) and ZYP1 (chromosome axes) in both WT and *Hvst1* was difficult to conduct due to abnormal synapsis in the mutant. Given the inability to accurately stage late prophase I in BW233 meiocytes due to the perturbation of SC dissolution we compared anthers of similar size as anther size is strongly correlated with meiotic progression (Arrieta *et al*., 2020). We found that for similarly sized anthers, WT Bowman displayed resolved MLH3 foci at pachytene while BW233 did not (Fig. S17), which is consistent with a delay in CO resolution (Colas *et al*., 2016). Co‐immunolocalization of MLH1 (Fig. S18) revealed a similar pattern to MLH3, with a large number of intermediates which persist at later stages. These intermediates converge into larger stretches as prophase I progresses but fail to resolve as discrete foci (Fig. S18f). The class I CO resolution marker HEI10 (Ziolkowski *et al*., 2017; Serra *et al*., 2018) typically loads on chromosome axes at zygotene (Fig. 6b) and co‐localizes with ZYP1 at pachytene (Fig. 6d) in WT Bowman. In BW233, although HEI10 maintains co‐localization with ZYP1, it does not form typical distinct foci but co‐localizes with the abnormal ZYP1 polycomplex‐like structure (Fig. 6f,h).

**Fig. 7:**
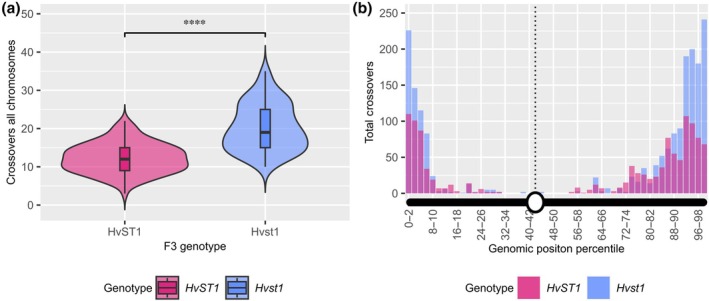
Hordeum *vulgare Hvst1* mutants show a significant increase in distal crossover (CO) frequency in all seven chromosomes. Comparison between the number and distribution of CO events detected using 50 K iSelect markers across all seven chromosomes between *Hvst1* (in Blue; *n* = 95) and *HvST1* (wild‐type (WT); in pink; *n* = 95). (a) Each violin represents the density of CO counts in F3 individuals carrying either *HvST1* (pink, WT allele) or *Hvst1* (blue) genotype. The embedded boxplots show the median and interquartile range (IQR) range. Hvst1 individuals exhibited significantly higher CO frequencies compared with HvST1 (Welch's *t*‐test; ****P < 2.2 × 10^−16^). The bimodal distribution observed for HvST1 indicates heterogeneity in recombination rates within this genotype. (b) A histogram of COs observed in all seven chromosomes binned in 2% intervals by genomic position (Mbp) relative to the Morex V3 chromosome assembly length, with the average position of the centromere marked by a dotted line and *x*‐axis illustration of a generic chromosome. This shows that the increase in the overall number of COs in *Hvst1* compared with WT is predominantly driven by an increase in COs towards chromosome ends (largely the first 10% of the chromosome length in Mbp).

Due to the stickiness of *Hvst1* chromosomes, the unclear MLH3 labelling and the HEI10 polycomplex, it was not possible to obtain accurate chiasma or MLH3/HEI10 foci counts from immunocytology of meiocytes. We therefore directly assessed COs in *Hvst1* mutant progeny by genetic analysis of 376 F_3_ plants derived from 24 F_2_ families selected from a cross between BW233 (*Hvst1*) x cv Barke (*HvST1*) after using marker‐assisted selection to identify genotypes homozygous for *HvST1* or *Hvst1*. We focused initially on Chromosomes 1H, 5H, and 6H using 48 KASPar markers (LGC Genomics). We observed that despite indicative observations in Metaphase I meiocytes the result of the *Hvst1* mutation was a slight increase in measurable COs in *Hvst1* populations compared with the WT F3 families in Chr1H and Chr5H (Fig. S19; *P* = 0.01; Wilcoxon rank sum test). To confirm that increased COs in *Hvst1* were genome‐wide, we then chose 95 of the 376 F_3_ plants and genotyped them using the barley 50K iSelect SNP array (Bayer *et al*., 2017). We calculated genome‐wide CO frequency after filtering for poorly mapped markers and F_2_ COs (Fig. S20). Total observed COs increased in *Hvst1* compared with *HvST1* across all chromosomes – primarily distally – by an average of 73% and up to 150% (Fig. 7; Table S9). This shows that the absence of HvST1 E3 ligase activity increased COs in those meiocytes that formed viable gametes.

### HvST1 is capable of ubiquitinating ASY1

**Fig. 8:**
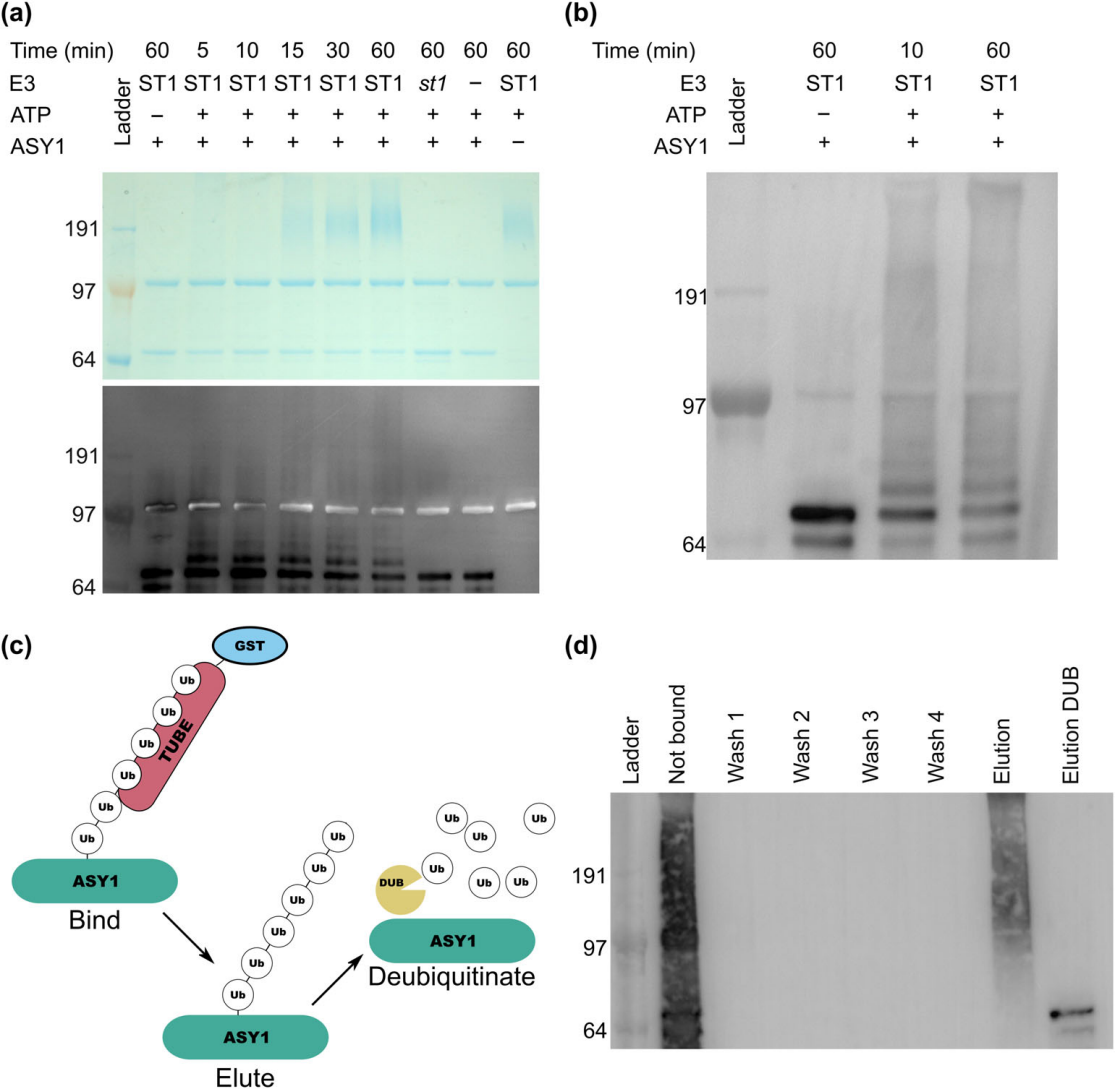
Hordeum *vulgare* HvST1 is capable of ubiquitinating ASY1. (a) Coomassie (above) and western blot (below) of HvST1‐ASY1 substrate ubiquitination assay. The western blot was probed first with anti‐histidine^6^ (negative representation, white signal), labelling ubiquitination cascade component proteins then with anti‐HvASY1 (positive representation, additional black signal). High‐molecular‐weight protein accumulates over time in all reactions in which functional HvST1 and the components of the ubiquitination cascade are present, but not in the absence of ATP, HvST1, or with nonfunctional Hvst1. Increase in the molecular weight of HvASY1 is observed over time where both a functional ubiquitination cascade involving HvST1 and HvASY1 are present. This indicates that HvST1 E3 is capable of ubiquitinating HvASY1, that this function is lost with the loss of the E2‐interacting RING domain in *Hvst1*, and that the anti‐ASY1 antibody does not bind polyubiquitinated protein or any other component of the reaction nonspecifically. (b) An anti‐HvASY1 only western blot showing an increase in HvASY1 molecular mass over time in combination with a functional HvST1 driven ubiquitination cascade but not in the absence of ATP to activate this cascade, illustrating ubiquitination of HvASY1 by HvST1 more clearly. (c) A diagrammatic representation of the GST‐TUBE capture experiment used to recover polyubiquitinated protein from the substrate ubiquitination assay and reverse ubiquitination using a broad‐spectrum DUB enzyme. The GST‐tagged TUBE construct binds polyubiquitin chains and the DUB enzyme breaks these chains into their ubiquitin monomers, returning the polyubiquitinated protein to its original molecular mass. (d) Western blot of fractions recovered from GST‐TUBE capture of polyubiquitinated products of HvST1‐HvASY1 substrate ubiquitination assay probed with anti‐HvASY1 antibody. HvASY1 is recovered by TUBE capture of ubiquitinated protein in the eluted fraction and upon treatment of this fraction with broad spectrum deubiquitinating (DUB) enzyme is returned to its original size (*c*. 64 KDa). This confirms that the increase in HvASY1 molecular mass in the substrate ubiquitination assay is the result of its polyubiquitination. For all western blots and the Coomassie‐stained PAGE, gel marker sizes in KDa are indicated on the left.

To determine whether abnormal synapsis observed in *Hvst1* mutants might be explained by failure of SC protein ubiquitination (required for their removal by the proteasome), we conducted an *in vitro* substrate ubiquitination assay with purified HvASY1 and HvST1 proteins. We found that HvASY1 is ubiquitinated in the presence of HvST1 and all other required components of the ubiquitination cascade (Fig. 8a,b), visible as an increase in mass on western blots probed using anti‐ASY1 antibody. No high molecular weight protein is labelled in anti‐ASY1 western blots in the absence of ATP, with purified Hvst1, or in the absence of HvASY1 (Fig. 8a), suggesting that the observed ubiquitination of HvASY1 is dependent on the HvST1 RING domain and the ubiquitination cascade. To confirm that high molecular weight HvASY1 was ubiquitinated, the polyubiquitinated products of this assay were captured using GST‐TUBE and treated with broad‐spectrum deubiquitinating enzyme which recovered HvASY1 at its original mass (Fig. 8c,d).

## Discussion

In plants, mammals, and budding yeast, the formation of COs and synapsis is tightly linked (Grey & de Massy, 2022). We have previously shown that mutations in barley meiotic genes dramatically affect meiosis and, in general, reduce COs (Colas *et al*., 2016, 2019), but also that mutations in conserved meiotic genes in barley (Colas *et al*., 2016, 2023) do not always lead to the expected phenotype when compared to other plants, including *Arabidopsis* (Jackson *et al*., 2006; Bleuyard & White, 2004) or rice (Ren *et al*., 2021).

Here, we have identified and characterized an E3 ubiquitin ligase that is highly expressed during meiosis, providing the first evidence of a unique ubiquitination pathway that regulates meiosis in barley. We have called this gene *HvST1* (*Sticky Telomeres 1*), due to the stickiness of chromosomes at Metaphase I that seemed to be in the telomere region. This stickiness made chiasma counts in *Hvst1* challenging, but we did find a significant increase in the average number of rod‐bivalent chromosomes and a decrease in ring bivalent chromosomes observed in *Hvst1* metaphase compared with the WT. We also observed a large variation in chiasma number in BW233 compared with the WT which in turn led to a significant, albeit small, decrease of chiasma in BW233 compared with the WT, reflecting different metaphase configurations.

The most significant phenotype of the *Hvst1* genotype was abnormal synapsis due to the formation of ZYP1 polycomplex‐like ‘clusters’ near the telomere region. Contrary to our initial hypothesis, we found that the initial formation of the telomere cluster was not altered in the mutant but that the telomeres tended to remain located at one side of the nucleus alongside ZYP1 clustering at the onset of delayed synapsis. Moreover, analysis of the chromatin state in BW233 using antibodies targeting methylated histones indicated an altered chromatin state, although proteomic analysis of histone methylation did not show statistically significant differences in any such modification. This suggests that HvST1 activity does not regulate the relative amount of histone methylation although it is still possible that loss of HvST1 function may affect the distribution of histone methylation along the chromatin length. Accordingly, the apparent reduction of chromatin compaction in BW233 is likely a reflection of delayed synapsis.

We also found that the altered distribution of ZYP1 in *Hvst1* constrained the distribution of HEI10 in late prophase, while HvDMC1 and MLH3 recruitment was unaltered, but they failed to resolve into discrete foci. The failure of MLH3 foci formation and concentration of HEI10 in the subtelomeric polycomplex also prohibited cytological counting of class I COs based on recombination intermediate foci in late prophase I. However, F3 50K genetic CO data showed a clear and substantial increase in the number of subtelomeric CO events in *Hvst1* relative to the WT. Further work is required to determine the pathway responsible for this increase in COs. Successful progeny included in the genetic CO data likely represent a bias towards less extreme male meiotic phenotypes exhibiting better chromosome alignment (Fig. S10) and proper segregation as these are more likely to develop to form fertile pollen. As such, the genetic CO data, while a direct reflection of the effective CO rate between generations, is an incomplete representation of cytological CO repair pathways. An increase in COs alongside a higher number of rod‐bivalent chromosomes at metaphase can be explained by a loss of obligate COs alongside COs which occur closer together which can be counted as a single chiasma. Underprediction of CO counts from the analysis of chromosomes at metaphase has been reported in the wheat *fancm* mutant (Desjardins *et al*., 2022) as well as the *Arabidopsis zyp1* (France *et al*., 2021), and *asy1‐3* mutants (Lambing *et al*., 2020). The phenotype of *Hvst1* bears some other similarities to *zyp1* null mutants in *Arabidopsis*, including persistent chromosome interlocks and failure of ASY1 depletion (Capilla‐Pérez *et al*., 2021; France *et al*., 2021). This indicates that much of the *HvST1* phenotype might derive from downstream effects of the failure or severe delay in complete ZYP1 polymerization.

Polyubiquitination of HvASY1 by HvST1 *in vitro* provides an indication that the lack of ZYP1 polymerization observed in *Hvst1* may result from the loss of this post‐translational modification of ASY1. This observation supports the findings of Osman *et al*. (2018) who previously identified proteasomal and ubiquitination‐related proteins in association with ASY1 in a pulldown from *Brassica oleracea* and is consistent with the observed localization of ubiquitination to the chromosomal axes during meiotic prophase (Rao *et al*., 2017; Orr *et al*., 2021b). RING‐type E3 ubiquitin ligases confer specificity to the ubiquitination cascade by binding both E2‐conjugating enzymes, thereby increasing the reactivity of E2‐Ub conjugates, and substrate proteins, catalysing the direct transfer of ubiquitin to the substrate protein itself, or existing polyubiquitin chains on its surface (Dove *et al*., 2016; Iconomou & Saunders, 2016). The canonical fate of polyubiquitinated proteins is proteasomal degradation, although a range of substrate fates can arise from ubiquitination determined by polyubiquitin chain topology, which is itself largely driven by a preference for particular lysine residues on the part of the interacting E2s (Dove *et al*., 2016). Combining the loss of E3 ligase activity in the *Hvst1* mutant with the retention of ASY1 on the axis in late prophase I in BW233 (Fig. 3g,h), peak of *HvST1* expression in meiocytes at Pachytene–Diplotene (Fig. 2b), the co‐localization of pro‐CO factor HEI10 with ZYP1 during delayed BW233 ZYP1 polymerization (Fig. 6f,h), and the significant increase in distal CO events in homozygous *Hvst1* progeny in both KASP and iSelect 50 K analysis (Fig. 7), we propose a model for HvST1 function in barley meiosis (Fig. S21) in which ASY1 turnover in early prophase I is required for ZYP1 polymerization and by extension for normal SC formation, CO resolution, and interference. A requirement for lateral element ubiquitination and proteasomal degradation for synapsis progression has been previously demonstrated in mice where interaction of the SKP1‐Cullin‐F‐box (SCF) complex in conjunction with F‐box protein FBXO47 with the ASY1 orthologue HORMAD1 is required for normal progression of synapsis (Guan *et al*., 2022; Ma *et al*., 2024). While the increase in subtelomeric CO frequency in BW233 is accompanied by a decrease in peri‐centromeric CO frequency compared with the WT (Fig. 7), the CO frequency is significantly higher overall in BW233, meaning that the impact of the loss of HvST1 E3 ligase activity is not simply a redistribution of COs from peri‐centromeric to subtelomeric regions. The dosage of ASY1, and fellow HORMAD protein ASY3, along the chromosome axis in *Arabidopsis* and wheat are broadly inverse to typical CO patterning, following an ascending gradient from telomere to centromere (Lambing *et al*., 2020). In isolation this observation might be taken as an indication that ASY1 is a dose‐dependent suppressor of COs. However, reduced ASY1 dosage has been reported to lead to a reduction in overall CO frequency and a shift in CO distribution towards the subtelomeric region (Lambing *et al*., 2020; Di Dio *et al*., 2023). In wheat, reduced ASY1 dosage also resulted in incomplete ZYP1 polymerization leading to asynchronous meiotic progression that arrested at pachytene and diplotene (Di Dio, *et al*., 2023). ASY1 is clearly involved in the molecular mechanics governing CO number, proximity, and distribution but a reduction in ASY1 dosage (in *asy1* mutants) and the retention of ASY1 (in *Hvst1*) appear to have similar effects on ZYP1 polymerization, CO distribution, and CO interference although an inverse effect on overall CO frequency. This surprising series of observations likely reflects both the highly dynamic nature of the SC and the involvement of ASY1 in a number of distinct processes governing CO resolution throughout prophase I. ASY1 dosage is correlated with SPO11‐1 in chromosome arms and DMC1 dynamics are disrupted in *asy1* (Lambing *et al*., 2020). The reduction in CO frequency with reduced ASY1 dosage might therefore result from a reduction in the formation of DSBs and perturbed stabilization of strand exchange intermediates. Axes retention of ASY1, by contrast, may promote these early events while similarly constraining the diffusion of pro‐CO factors acting later in CO formation, such as HEI10.

In *Hvst1* mutants, HEI10 distribution is clearly constrained by altered ZYP1 distribution resulting in a localized increase in HEI10 concentration and long stretches of HEI10 in partially synapsed regions as opposed to discrete foci. It has been demonstrated that the distribution of HEI10 is constrained by ZYP1 – so long as ZYP1 is present – and that altered ZYP1 distribution can affect CO number and distribution (Capilla‐Pérez *et al*., 2021; France *et al*., 2021; Fozard *et al*., 2023). In this context, the observed increase in distal COs in *Hvst1* may be best understood within the proposed HEI10 coarsening model (Morgan *et al*., 2021). It has been demonstrated that an increase in HEI10 concentration results in a reduction in CO interference and an increase in the total number of COs (Ziolkowski *et al*., 2017). The HEI10 coarsening model proposes that class I CO number and distribution can largely be attributed to the concentration, SC restricted diffusion, and reduced rate of escape over time of HEI10 from larger foci at recombination intermediates which are then resolved as COs. However, altered synapsis in *Hvst1* mutants prohibited accurate cytological counting of class I or class II recombination intermediates (Figs 6, S17, S18), putting determination of the pathway responsible for the increase of COs observed in KASP marker and 50 K analysis outside the scope of this work.

Recent description of the rice *Osclr1*/*dsnp1* meiotic phenotype highlights several similar immunocytological observations, including the formation of a ZEP1 polycomplex, the ZYP1 equivalent in rice, and failure of PAIR2 depletion, the ASY1 equivalent in rice, indicating conserved meiotic function in grasses (Ren *et al*., 2021). However, the induced *dsnp1* mutation rendered these plants completely sterile, *Osdsnp1* metaphase spreads do not show the lack of chromosome condensation observed in *Hvst1*, and the ZEP1 polycomplex does not appear to retain subtelomeric localization as in *Hvst1* (Ren *et al*., 2021). The authors also reported a reduction in class I COs based on reduced chiasmata counts in *Osdsnp1* compared with the WT and decreased HEI10 foci counts in an *Osdsnp1/zep1* double mutant background when compared to both the WT and *zep1* single mutant (Ren *et al*., 2021). The induced mutation in *Osdsnp1* is highly similar to *Hvst1* occurring early in the RING domain and presumably resulting in the loss of E3 ligase activity (Ren *et al*., 2021). Although OsDSNP1/OsCLR1 has a reported gain of function in response to heat and drought stress which is not conserved in other grasses (Park *et al*., 2019), indicating some evolutionary divergence, it does not seem likely that the function of this protein is meaningfully divergent from HvST1 in meiosis. The reported reduction in *Osdsnp1* chiasma may reflect the greater proximity of COs we observe in genetic CO data, resulting in inaccurate counts as described by Desjardins *et al*. (2022). The slight reduction of HEI10 foci in the *Osdsnp1/zep1* double mutant background compared with the WT might indicate that the increase in COs we observe in *Hvst1* is due to class II CO resolution, or that the altered ZYP1 environment and partial synapsis are essential to the increase in COs observed in *Hvst1*. The comparatively large reduction in HEI10 foci in *Osdsnp1/zep1* compared with the *zep1* single mutant is intriguing, possibly indicating a role for HvST1 in regulating class I CO resolution beyond its impact on ZYP1 polymerization, whether through ASY1 ubiquitination or other targets. Complete sterility and the distinct ZEP1 polycomplex behaviour in *Osdsnp1* most likely reflect fundamental differences in genome size, chromatin organization, and timing during meiosis between rice and barley. Further work is required to identify and validate the targets of HvST1 E3 ligase activity *in vivo* and to elucidate the protein–protein interactions underlying the *Hvst1* meiotic phenotype.

There is increasing interest in methods of altering the meiotic recombination landscape in plants through targeting of post‐translational modifications and the use of methods, such as virus‐induced gene silencing (VIGS), to downregulate CO suppressive genes in order to reduce the time, resources, and emissions associated with generating novel crop varieties with desirable traits (Desjardins *et al*., 2020; Raz *et al*., 2021). Further investigation of the molecular mechanics of *HvST1* might reveal new pathways and additional targets for such approaches. Similarly, identifying alternative mutations in *HvST1*, its promoter, targets, or proteins regulating its activity might result in a similar increase in COs without the same degree of semi‐sterility, which would enhance its use in plant breeding. While it is presently unclear exactly how subtelomeric COs are increased in *Hvst1*, this mutation could find immediate practical application in breeding programmes to increase meiotic recombination – in particular, between chromosomes of distantly related genotypes that are being exploited as a source of novel traits, such as disease, heat, and drought resistance and to disrupt stubborn linkage drag. Serra *et al*. (2018) demonstrated that combining *HEI10* overexpression with mutation of *recq4*, an inhibitor of class II COs, led to an additive increase in COs in Arabidopsis. Combining *Hvst1* with the recently described *Hvrecql4* mutation (Arrieta *et al*., 2021) may also lead to such an additive effect and may provide further insight into the pathway leading to the observed increase in COs in *Hvst1*.

## Competing interests

None declared.

## Author contributions

IC, LR, JNO, SUM and RW designed the experiment. SUM, JNO, DL, MM, AB and NM conducted the experiments and data analyses. JNO, RW and IC wrote the manuscript. All authors reviewed the manuscript. JNO and SUM contributed equally to this work.

## Disclaimer

The New Phytologist Foundation remains neutral with regard to jurisdictional claims in maps and in any institutional affiliations.

## Supporting information


**Fig. S1** Comparison of meiosis stages of Bowman (*HvST1*) and BW233 (*Hvst1*).
**Fig. S2** The diversity of BW233 metaphase configuration.
**Fig. S3** Chiasma counts from Bowman and BW233 metaphase spreads and interlocks at Metaphase I and Anaphase I.
**Fig. S4** Pipeline for *des12.w* fine mapping.
**Fig. S5** *des12.w m*utation identification.
**Fig. S6** Fertility of the allelic test crosses and their parental lines.
**Fig. S7** Clipped Sanger sequencing chromatograms for *des12*, *des4*, and *des1* alleles.
**Fig. S8** CRISPR/*cas9* Line for *HvST1*.
**Fig. S9** Maximum likelihood phylogeny of *HvST1*.
**Fig. S10** Amino acid alignment of *HvST1*, its orthologues in the Poaceae, and *Arabidopsis thaliana CIP8*.
**Fig. S11** Boxplots of *HvST1* expression from the EoRNA database under drought, heat, and salt stress conditions and in all tissues.
**Fig. S12** MS/MS confirmation of autoubiquitination assay Coomassie band identities.
**Fig. S13** E2 scan plate HvST1 vs *Hvst1*.
**Fig. S14** Immunocytology of late synapsis in spontaneous *Hvst1* mutants.
**Fig. S15** Synapsis of induced *HvST1* mutants.
**Fig. S16** Boxplot of histone methylation modification in Bowman (*HvST1*), and BW233 (*Hvst1*).
**Fig. S17** MLH3 behaviour in Bowman (*HvST1*), and BW233 (*Hvst1*).
**Fig. S18** Behaviour of MLH1 during synapsis.
**Fig. S19** Preliminary F3 KASP assay crossovers.
**Fig. S20** Recombination data filtering.
**Fig. S21** A proposed model of the role of HvST1 in synapsis.

**Table S1** KASP primers used to refine the des12.w interval.
**Table S2** Resequencing primers used to identify polymorphisms for marker development.
**Table S3** Markers used in preliminary KASP recombination analysis.
**Table S4** Primers for cloning MLOC_4107/HORVU7Hr1G092570 cDNA.
**Table S5** Gateway® primers for cloning MLOC_4107/HORVU7Hr1G092570 coding sequence into *Escherichia coli* expression vector pDEST‐HisMBP.
**Table S6** CRISPR/Cas9 synthetic guide RNA (sgRNA) oligonucleotides targeting MLOC_4107/HORVU7Hr1G092570.
**Table S7** Primers for genotyping CRISPR/Cas9 knockout plants.
**Table S8** Histone methylation summary.
**Table S9** Change in total crossovers observed per chromosome in WT and BW233 in 50K iSelect SNP chip data.
Please note: Wiley is not responsible for the content or functionality of any Supporting Information supplied by the authors. Any queries (other than missing material) should be directed to the *New Phytologist* Central Office.

## Data Availability

Barley 50K iSelect recombination data are available on FigShare (https://doi.org/10.6084/m9.figshare.21427953.v1). Code used in data analysis and plotting for this study is available at https://github.com/BioJNO/HvST1.
