# Supplementary material for: Loss of E3 ligase HvST1 function substantially increases distal crossover frequency
Source: New Phytol. 2025 Nov 30;249(3):1325–41. doi: 10.1111/nph.70757 (PMC12780313; doi:10.1111/nph.70757)
Supplement: Supplementary file 1 — Fig. S1 Comparison of meiosis stages of Bowman (HvST1) and BW233 (Hvst1). Fig. S2 The diversity of BW233 metaphase configuration. Fig. S3 Chiasma counts from Bowman and BW233 metaphase spreads and interlocks at Metaphase I and Anaphase I. Fig. S4 Pipeline for des12.w fine mapping. Fig. S5 des12.w mutation identification. Fig. S6 Fertility of the allelic test crosses and their parental lines. Fig. S7 Clipped Sanger sequencing chromatograms for des12, des4, and des1 alleles. Fig. S8 CRISPR/cas9 Line for HvST1. Fig. S9 Maximum likelihood phylogeny of HvST1. Fig. S10 Amino acid alignment of HvST1, its orthologues in the Poaceae, and Arabidopsis thaliana CIP8. Fig. S11 Boxplots of HvST1 expression from the EoRNA database under drought, heat, and salt stress conditions and in all tissues. Fig. S12 MS/MS confirmation of autoubiquitination assay Coomassie band identities. Fig. S13 E2 scan plate HvST1 vs Hvst1. Fig. S14 Immunocytology of late synapsis in spontaneous Hvst1 mutants. Fig. S15 Synapsis of induced HvST1 mutants. Fig. S16 Boxplot of histone methylation modification in Bowman (HvST1), and BW233 (Hvst1). Fig. S17 MLH3 behaviour in Bowman (HvST1), and BW233 (Hvst1). Fig. S18 Behaviour of MLH1 during synapsis. Fig. S19 Preliminary F3 KASP assay crossovers. Fig. S20 Recombination data filtering. Fig. S21 A proposed model of the role of HvST1 in synapsis. [file NPH-249-1325-s001.pptx]

## Slide 1
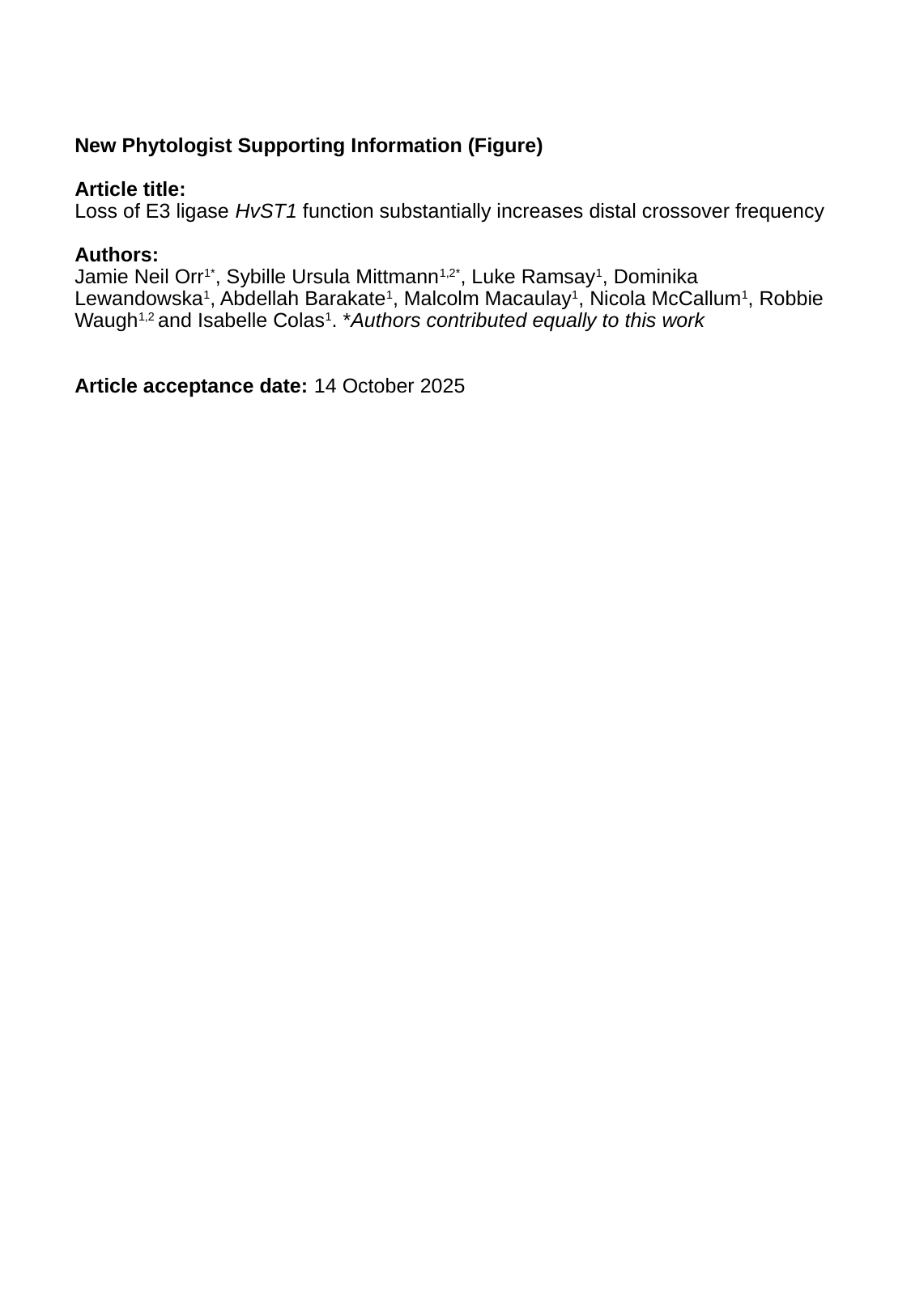

# New Phytologist Supporting Information (Figure) Article title: Loss of E3 ligase HvST1 function substantially increases distal crossover frequency Authors: Jamie Neil Orr1*, Sybille Ursula Mittmann1,2*, Luke Ramsay1, Dominika Lewandowska1, Abdellah Barakate1, Malcolm Macaulay1, Nicola McCallum1, Robbie Waugh1,2 and Isabelle Colas1. *Authors contributed equally to this work   Article acceptance date: 14 October 2025

## Slide 2
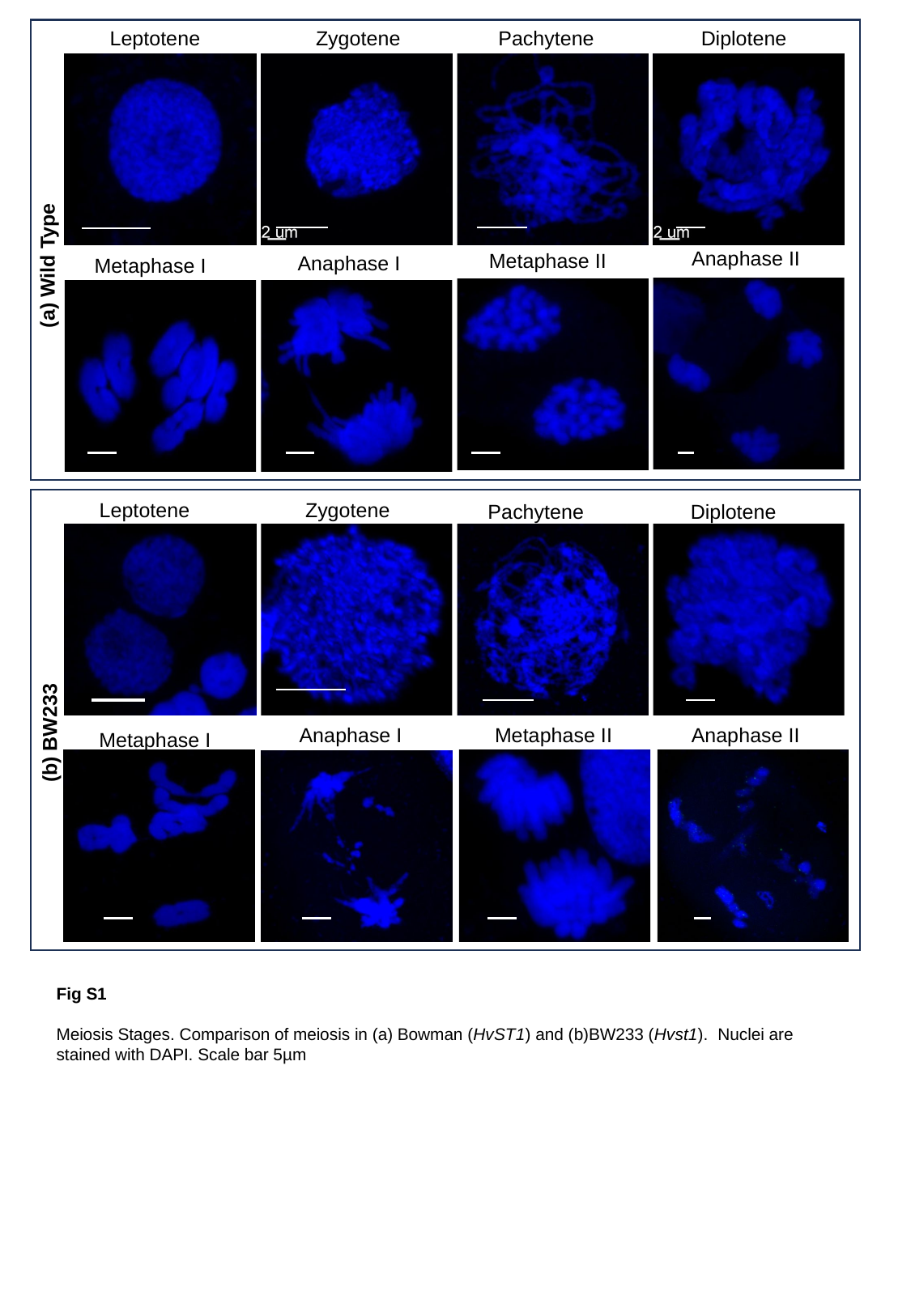

Zygotene
Pachytene
Leptotene
Diplotene
Anaphase II
Metaphase II
Anaphase I
(a) Wild Type
Metaphase I
Zygotene
Leptotene
Pachytene
Diplotene
(b) BW233
Metaphase II
Anaphase I
Anaphase II
Metaphase I
Fig S1
Meiosis Stages. Comparison of meiosis in (a) Bowman (HvST1) and (b)BW233 (Hvst1). Nuclei are stained with DAPI. Scale bar 5µm

## Slide 3
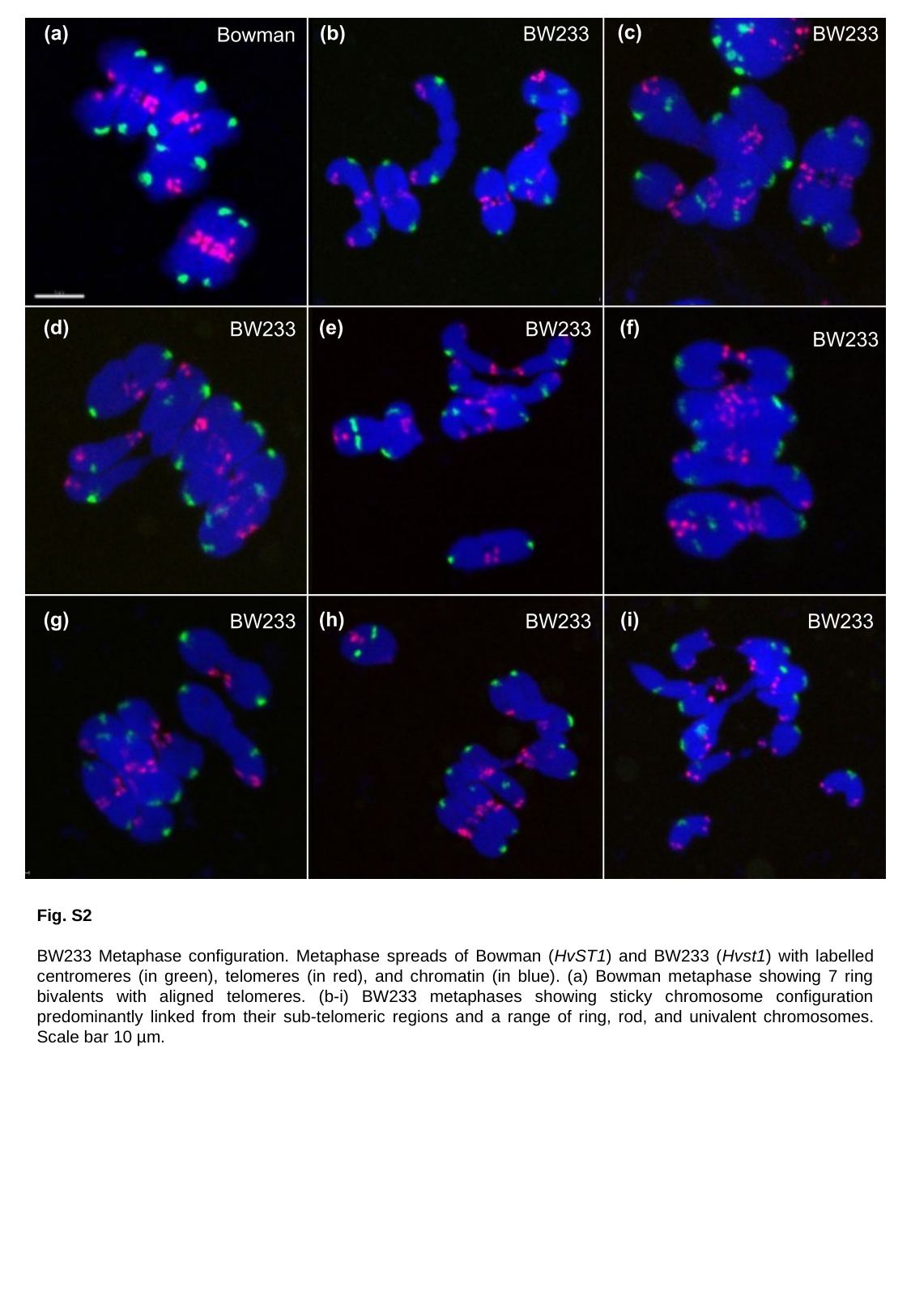

Fig. S2
BW233 Metaphase configuration. Metaphase spreads of Bowman (HvST1) and BW233 (Hvst1) with labelled centromeres (in green), telomeres (in red), and chromatin (in blue). (a) Bowman metaphase showing 7 ring bivalents with aligned telomeres. (b-i) BW233 metaphases showing sticky chromosome configuration predominantly linked from their sub-telomeric regions and a range of ring, rod, and univalent chromosomes. Scale bar 10 µm.

## Slide 4
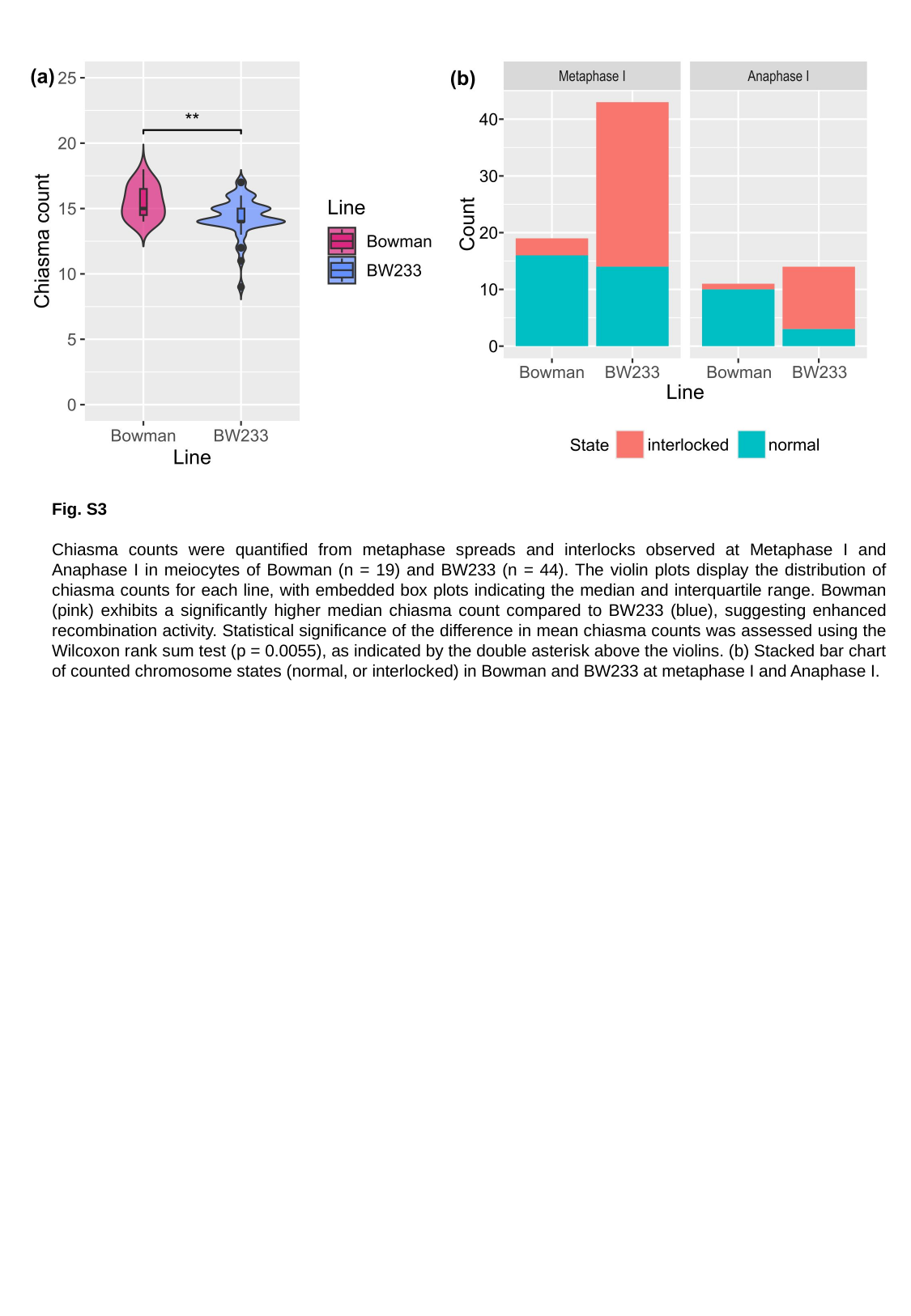

Fig. S3
Chiasma counts were quantified from metaphase spreads and interlocks observed at Metaphase I and Anaphase I in meiocytes of Bowman (n = 19) and BW233 (n = 44). The violin plots display the distribution of chiasma counts for each line, with embedded box plots indicating the median and interquartile range. Bowman (pink) exhibits a significantly higher median chiasma count compared to BW233 (blue), suggesting enhanced recombination activity. Statistical significance of the difference in mean chiasma counts was assessed using the Wilcoxon rank sum test (p = 0.0055), as indicated by the double asterisk above the violins. (b) Stacked bar chart of counted chromosome states (normal, or interlocked) in Bowman and BW233 at metaphase I and Anaphase I.

## Slide 5
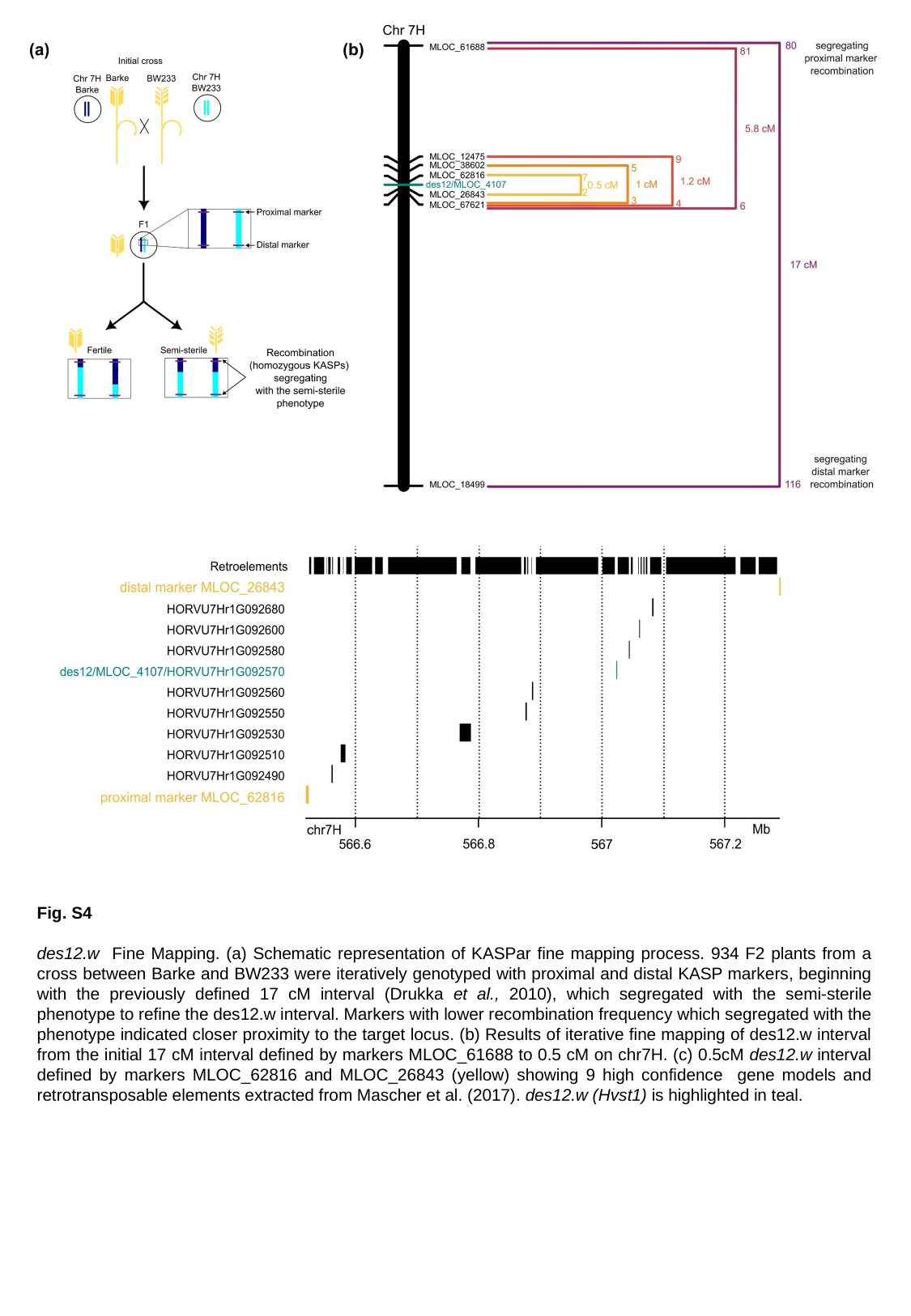

Fig. S4
des12.w Fine Mapping. (a) Schematic representation of KASPar fine mapping process. 934 F2 plants from a cross between Barke and BW233 were iteratively genotyped with proximal and distal KASP markers, beginning with the previously defined 17 cM interval (Drukka et al., 2010), which segregated with the semi-sterile phenotype to refine the des12.w interval. Markers with lower recombination frequency which segregated with the phenotype indicated closer proximity to the target locus. (b) Results of iterative fine mapping of des12.w interval from the initial 17 cM interval defined by markers MLOC_61688 to 0.5 cM on chr7H. (c) 0.5cM des12.w interval defined by markers MLOC_62816 and MLOC_26843 (yellow) showing 9 high confidence gene models and retrotransposable elements extracted from Mascher et al. (2017). des12.w (Hvst1) is highlighted in teal.

## Slide 6
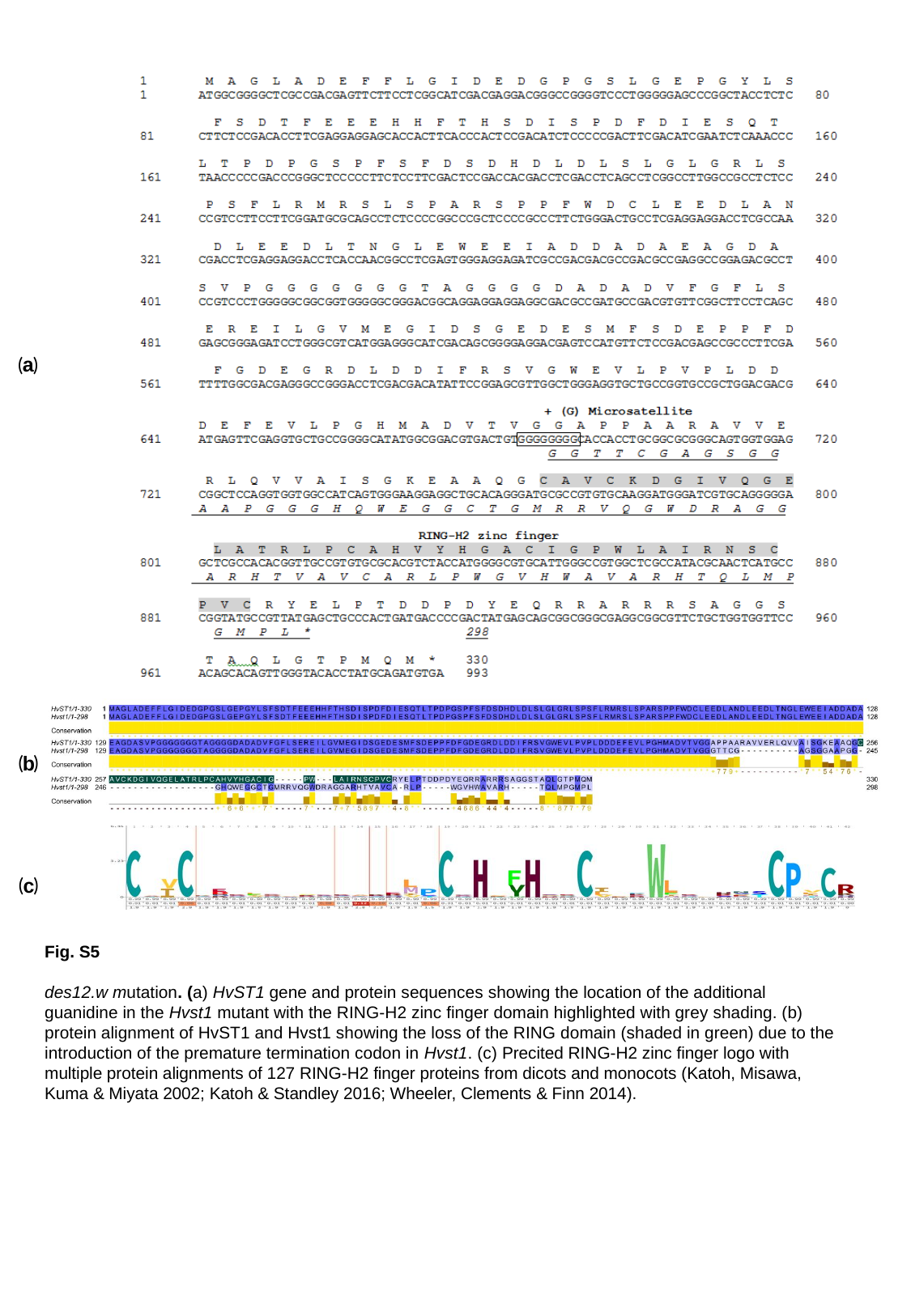

( )
a
( )
b
( )
c
Fig. S5
des12.w mutation. (a) HvST1 gene and protein sequences showing the location of the additional guanidine in the Hvst1 mutant with the RING-H2 zinc finger domain highlighted with grey shading. (b) protein alignment of HvST1 and Hvst1 showing the loss of the RING domain (shaded in green) due to the introduction of the premature termination codon in Hvst1. (c) Precited RING-H2 zinc finger logo with multiple protein alignments of 127 RING-H2 finger proteins from dicots and monocots (Katoh, Misawa, Kuma & Miyata 2002; Katoh & Standley 2016; Wheeler, Clements & Finn 2014).

## Slide 7
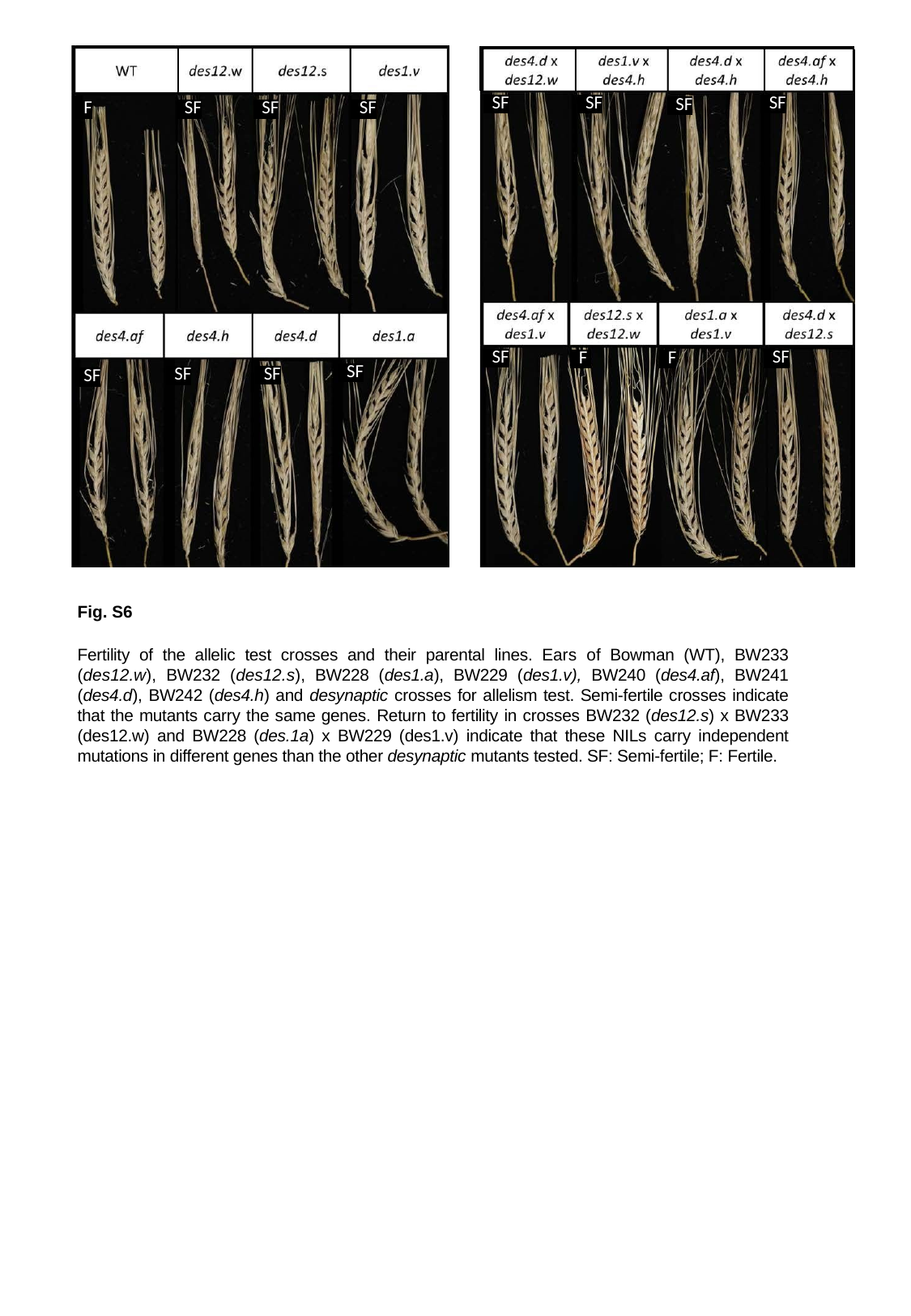

SF
 SF
 SF
 F
 SF
 SF
 SF
 SF
 SF
 SF
 SF
 SF
 SF
 SF
 F
 F
Fig. S6
Fertility of the allelic test crosses and their parental lines. Ears of Bowman (WT), BW233 (des12.w), BW232 (des12.s), BW228 (des1.a), BW229 (des1.v), BW240 (des4.af), BW241 (des4.d), BW242 (des4.h) and desynaptic crosses for allelism test. Semi-fertile crosses indicate that the mutants carry the same genes. Return to fertility in crosses BW232 (des12.s) x BW233 (des12.w) and BW228 (des.1a) x BW229 (des1.v) indicate that these NILs carry independent mutations in different genes than the other desynaptic mutants tested. SF: Semi-fertile; F: Fertile.

## Slide 8
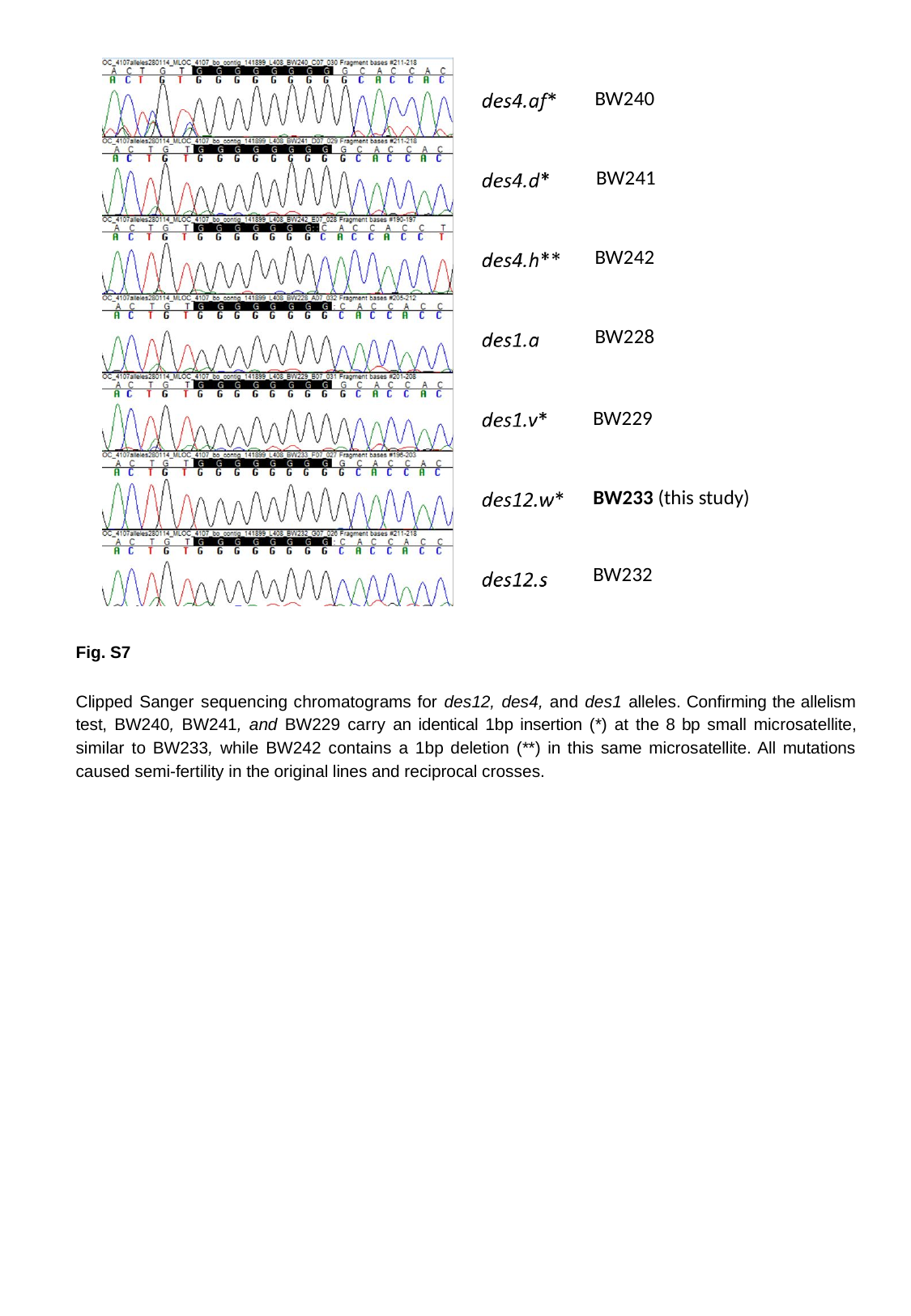

BW240
BW241
BW242
BW228
BW229
BW233 (this study)
BW232
Fig. S7
Clipped Sanger sequencing chromatograms for des12, des4, and des1 alleles. Confirming the allelism test, BW240, BW241, and BW229 carry an identical 1bp insertion (*) at the 8 bp small microsatellite, similar to BW233, while BW242 contains a 1bp deletion (**) in this same microsatellite. All mutations caused semi-fertility in the original lines and reciprocal crosses.

## Slide 9
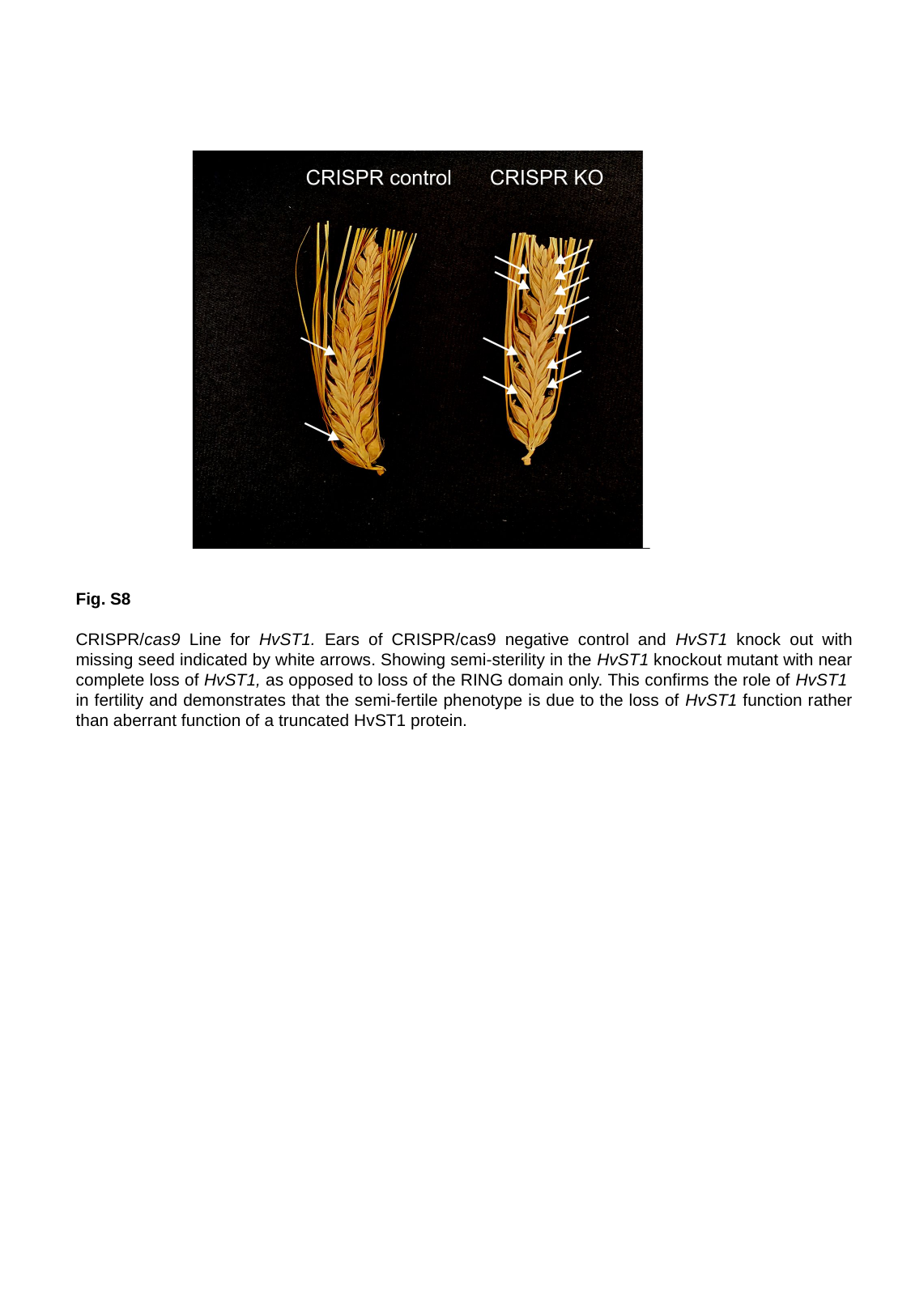

Fig. S8
CRISPR/cas9 Line for HvST1. Ears of CRISPR/cas9 negative control and HvST1 knock out with missing seed indicated by white arrows. Showing semi-sterility in the HvST1 knockout mutant with near complete loss of HvST1, as opposed to loss of the RING domain only. This confirms the role of HvST1 in fertility and demonstrates that the semi-fertile phenotype is due to the loss of HvST1 function rather than aberrant function of a truncated HvST1 protein.

## Slide 10
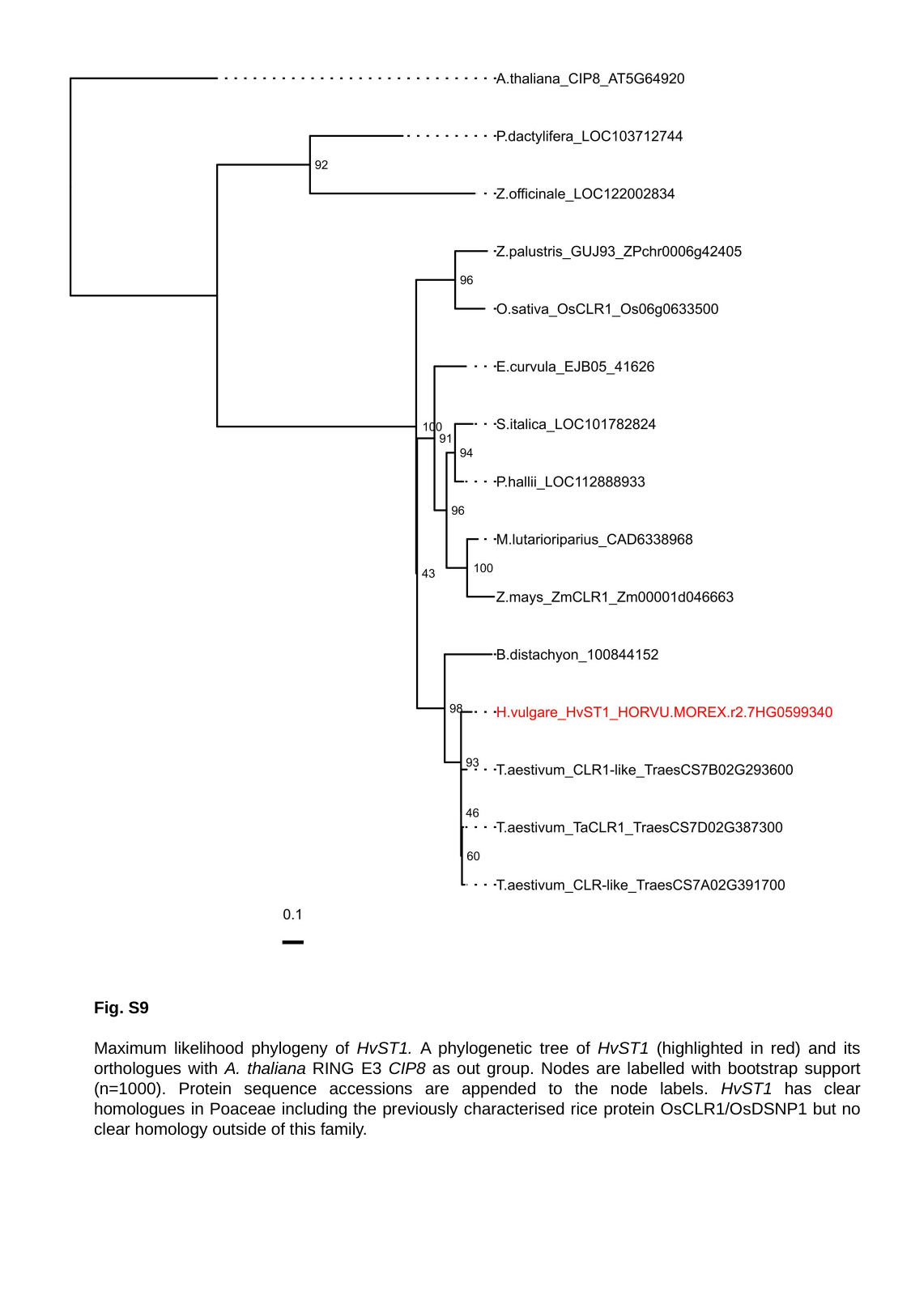

Fig. S9
Maximum likelihood phylogeny of HvST1. A phylogenetic tree of HvST1 (highlighted in red) and its orthologues with A. thaliana RING E3 CIP8 as out group. Nodes are labelled with bootstrap support (n=1000). Protein sequence accessions are appended to the node labels. HvST1 has clear homologues in Poaceae including the previously characterised rice protein OsCLR1/OsDSNP1 but no clear homology outside of this family.

## Slide 11
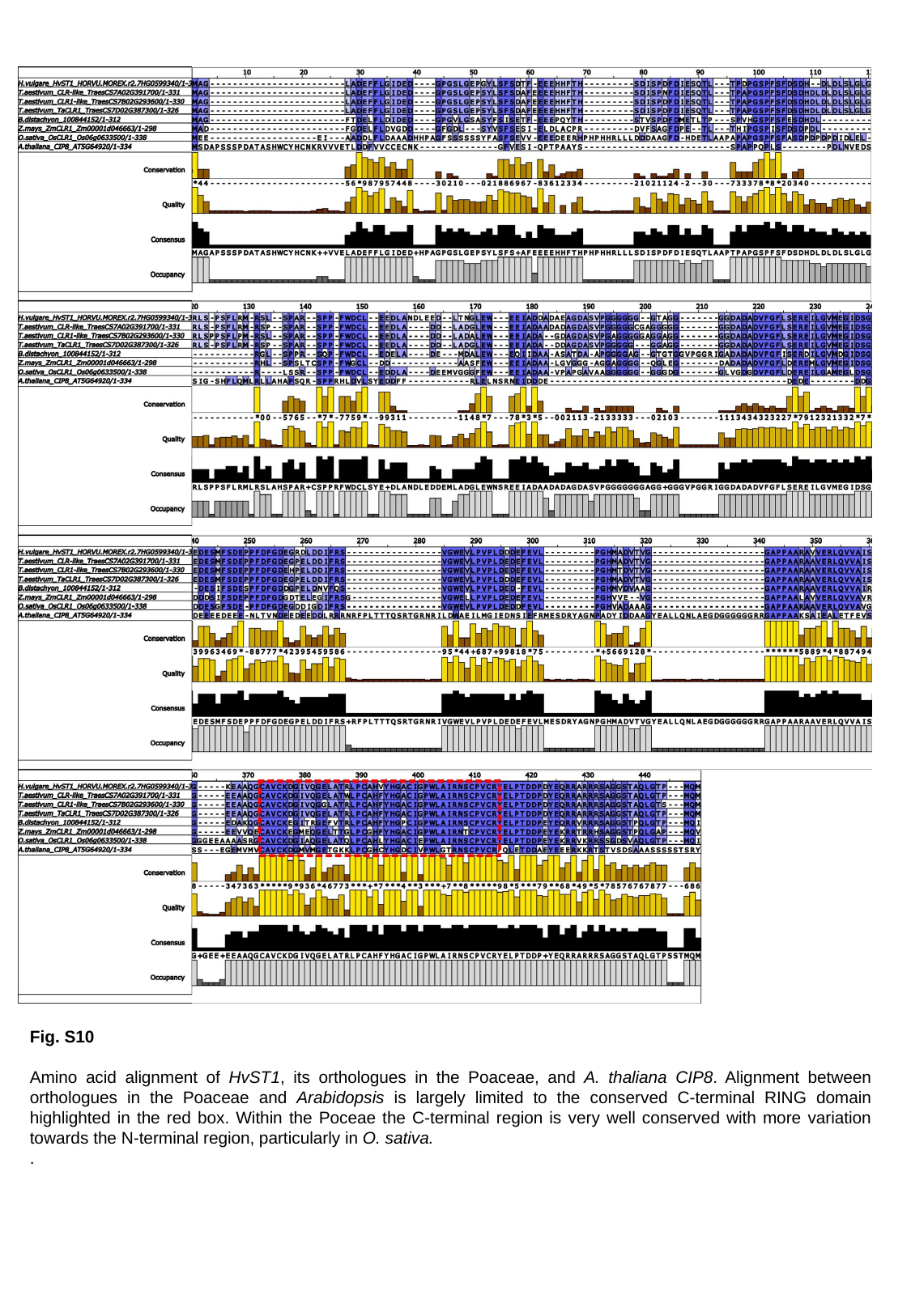

Fig. S10
Amino acid alignment of HvST1, its orthologues in the Poaceae, and A. thaliana CIP8. Alignment between orthologues in the Poaceae and Arabidopsis is largely limited to the conserved C-terminal RING domain highlighted in the red box. Within the Poceae the C-terminal region is very well conserved with more variation towards the N-terminal region, particularly in O. sativa.
.

## Slide 12
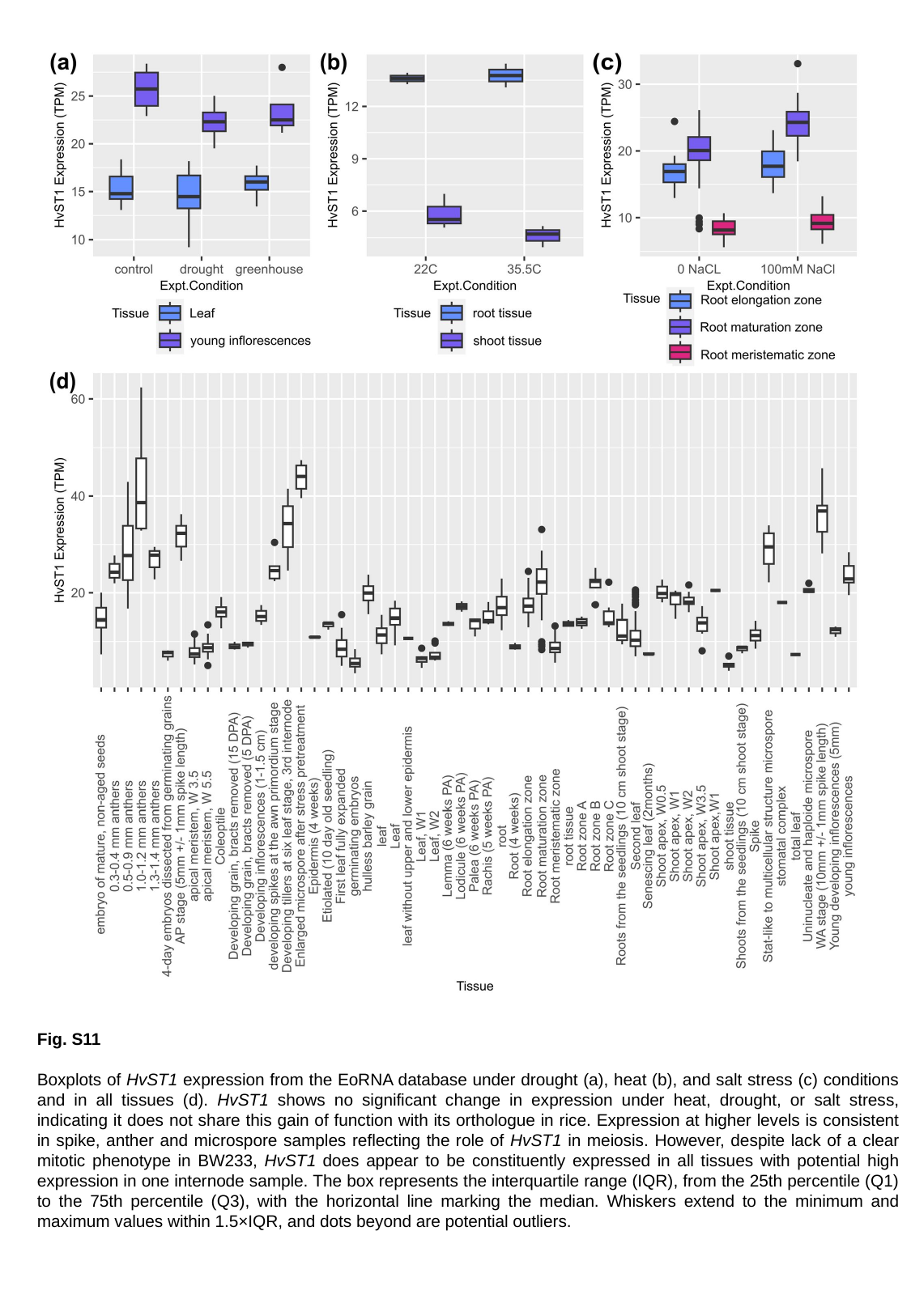

Fig. S11
Boxplots of HvST1 expression from the EoRNA database under drought (a), heat (b), and salt stress (c) conditions and in all tissues (d). HvST1 shows no significant change in expression under heat, drought, or salt stress, indicating it does not share this gain of function with its orthologue in rice. Expression at higher levels is consistent in spike, anther and microspore samples reflecting the role of HvST1 in meiosis. However, despite lack of a clear mitotic phenotype in BW233, HvST1 does appear to be constituently expressed in all tissues with potential high expression in one internode sample. The box represents the interquartile range (IQR), from the 25th percentile (Q1) to the 75th percentile (Q3), with the horizontal line marking the median. Whiskers extend to the minimum and maximum values within 1.5×IQR, and dots beyond are potential outliers.

## Slide 13
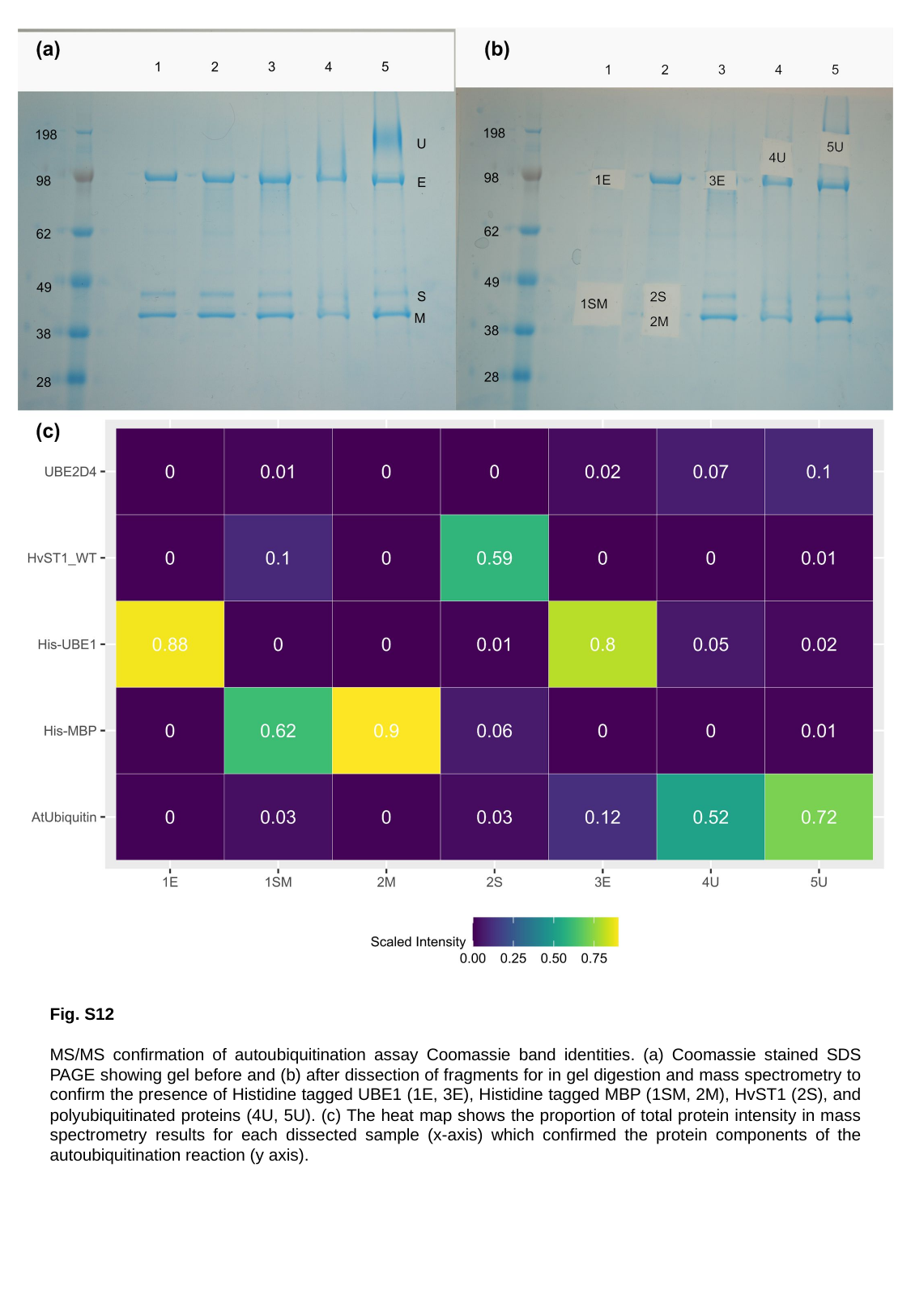

Fig. S12
MS/MS confirmation of autoubiquitination assay Coomassie band identities. (a) Coomassie stained SDS PAGE showing gel before and (b) after dissection of fragments for in gel digestion and mass spectrometry to confirm the presence of Histidine tagged UBE1 (1E, 3E), Histidine tagged MBP (1SM, 2M), HvST1 (2S), and polyubiquitinated proteins (4U, 5U). (c) The heat map shows the proportion of total protein intensity in mass spectrometry results for each dissected sample (x-axis) which confirmed the protein components of the autoubiquitination reaction (y axis).

## Slide 14
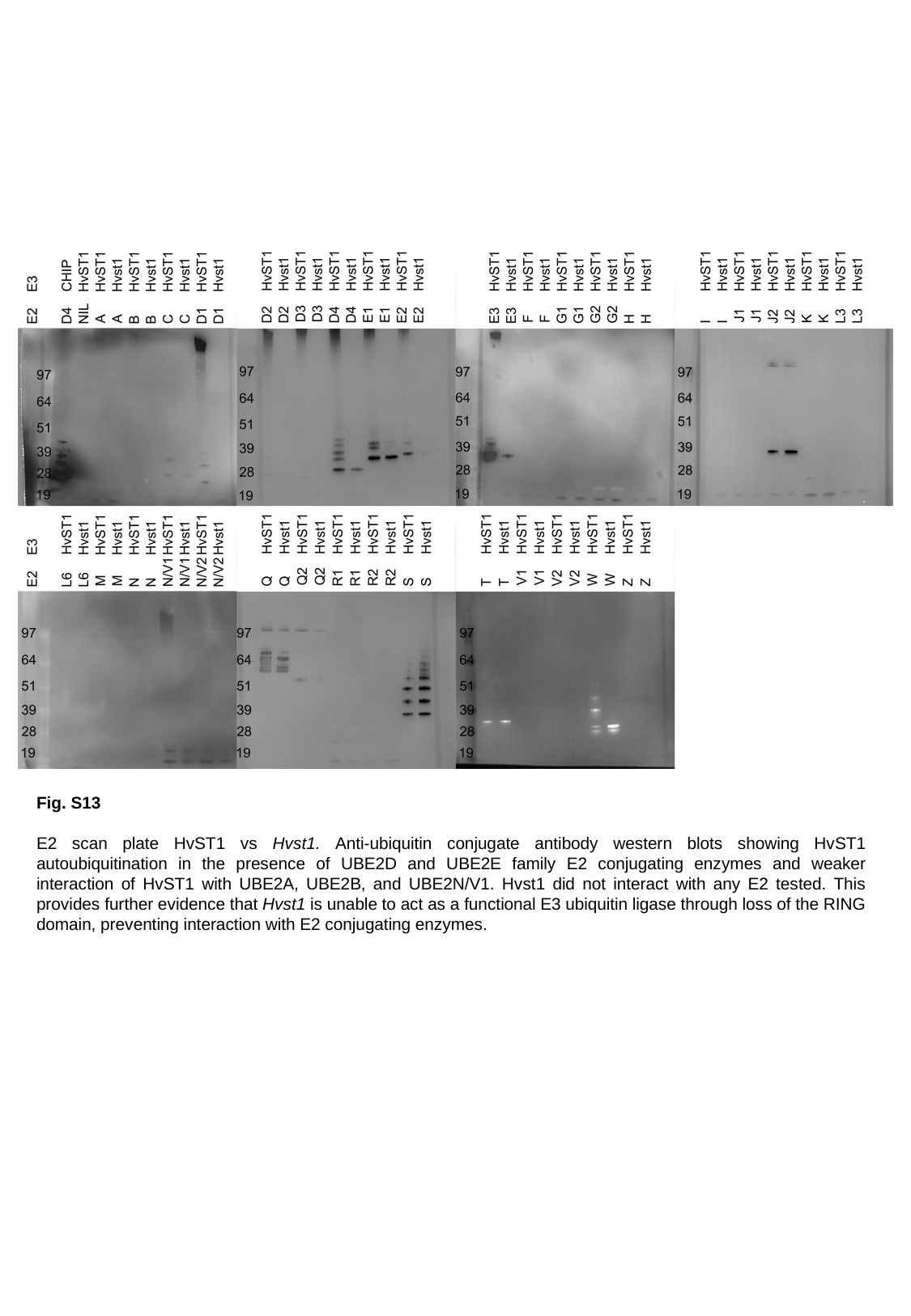

Fig. S13
E2 scan plate HvST1 vs Hvst1. Anti-ubiquitin conjugate antibody western blots showing HvST1 autoubiquitination in the presence of UBE2D and UBE2E family E2 conjugating enzymes and weaker interaction of HvST1 with UBE2A, UBE2B, and UBE2N/V1. Hvst1 did not interact with any E2 tested. This provides further evidence that Hvst1 is unable to act as a functional E3 ubiquitin ligase through loss of the RING domain, preventing interaction with E2 conjugating enzymes.

## Slide 15
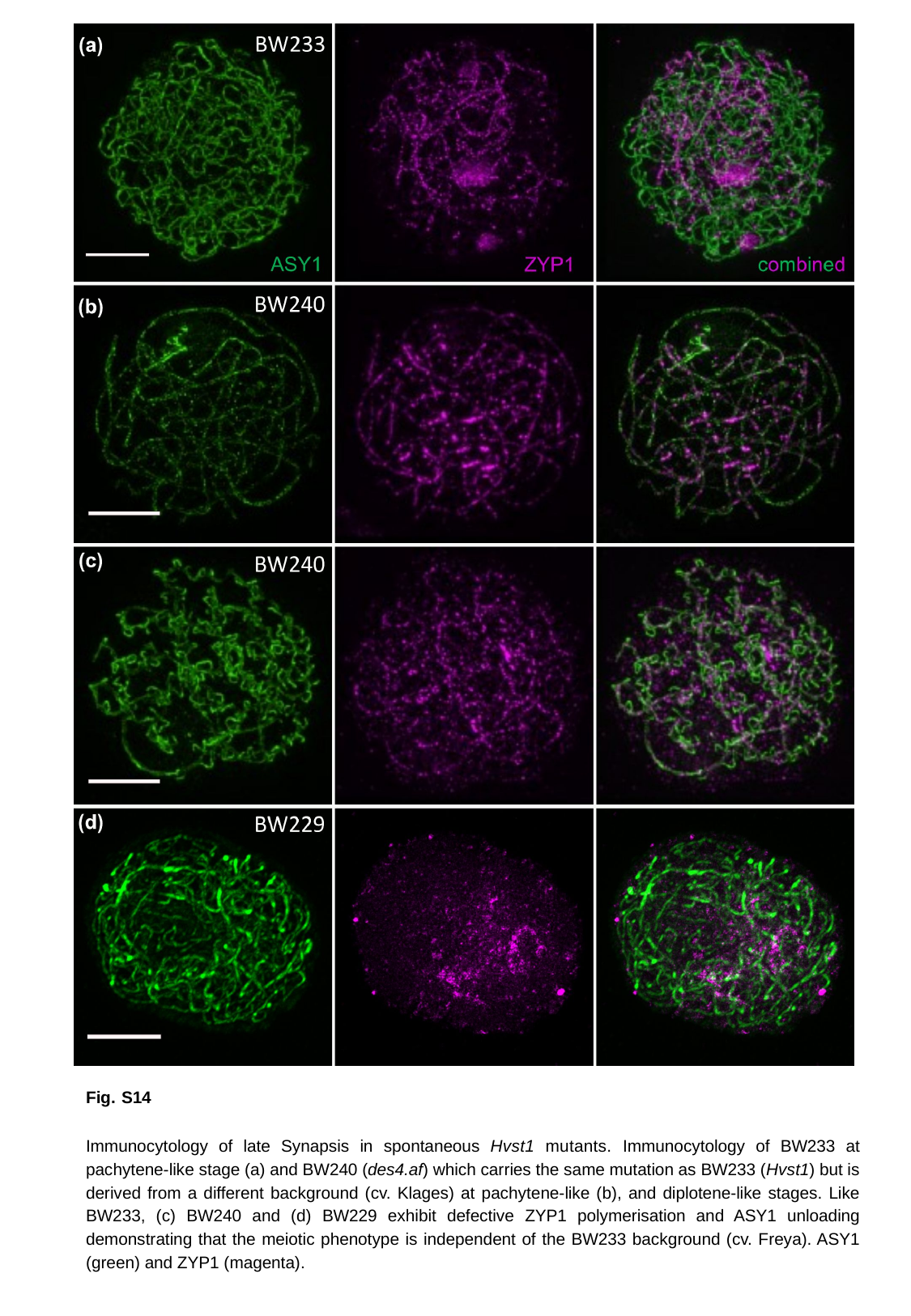

( )
( )
( )
( )
Fig. S14
Immunocytology of late Synapsis in spontaneous Hvst1 mutants. Immunocytology of BW233 at pachytene-like stage (a) and BW240 (des4.af) which carries the same mutation as BW233 (Hvst1) but is derived from a different background (cv. Klages) at pachytene-like (b), and diplotene-like stages. Like BW233, (c) BW240 and (d) BW229 exhibit defective ZYP1 polymerisation and ASY1 unloading demonstrating that the meiotic phenotype is independent of the BW233 background (cv. Freya). ASY1 (green) and ZYP1 (magenta).

## Slide 16
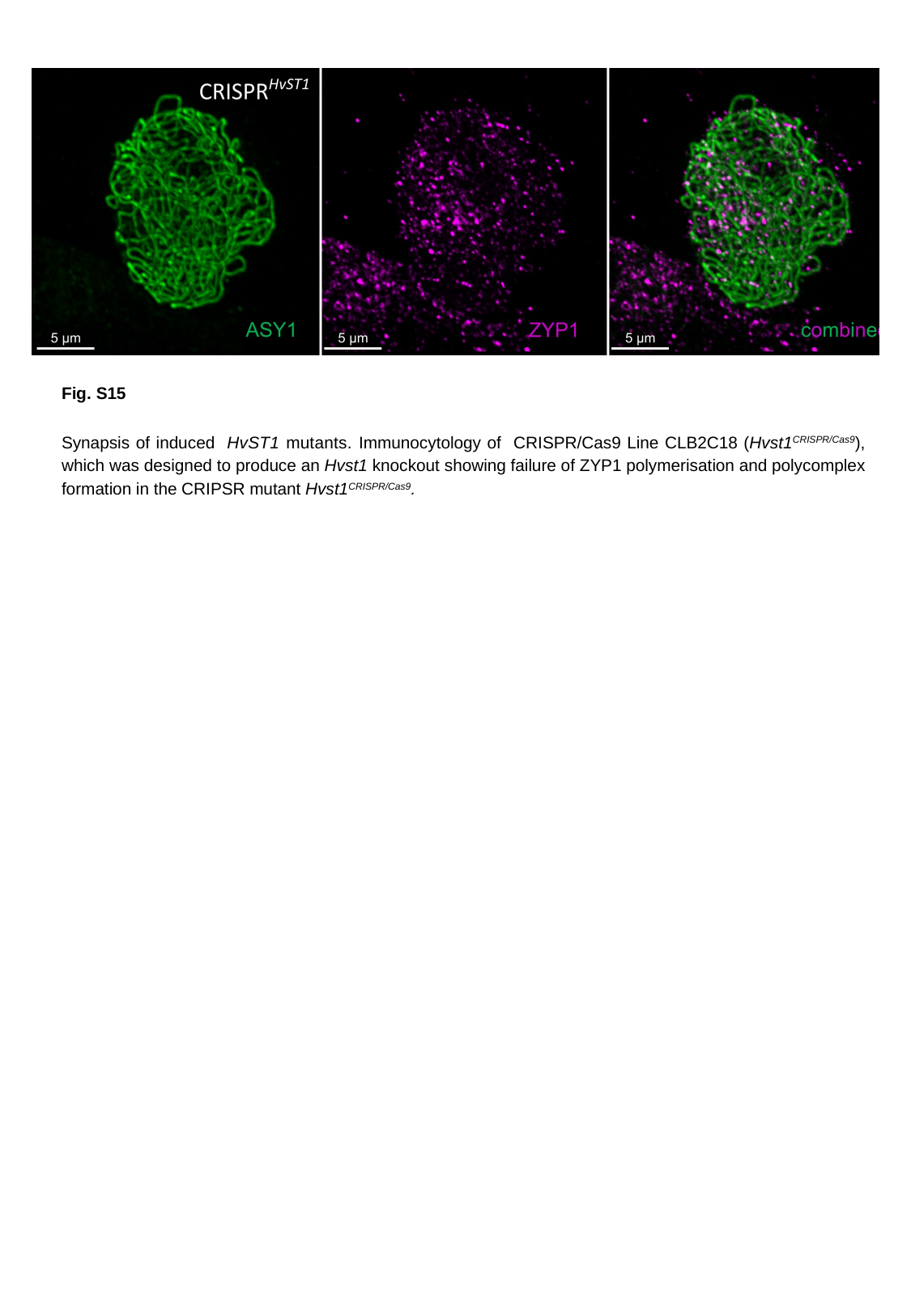

Fig. S15
Synapsis of induced HvST1 mutants. Immunocytology of CRISPR/Cas9 Line CLB2C18 (Hvst1CRISPR/Cas9), which was designed to produce an Hvst1 knockout showing failure of ZYP1 polymerisation and polycomplex formation in the CRIPSR mutant Hvst1CRISPR/Cas9.

## Slide 17
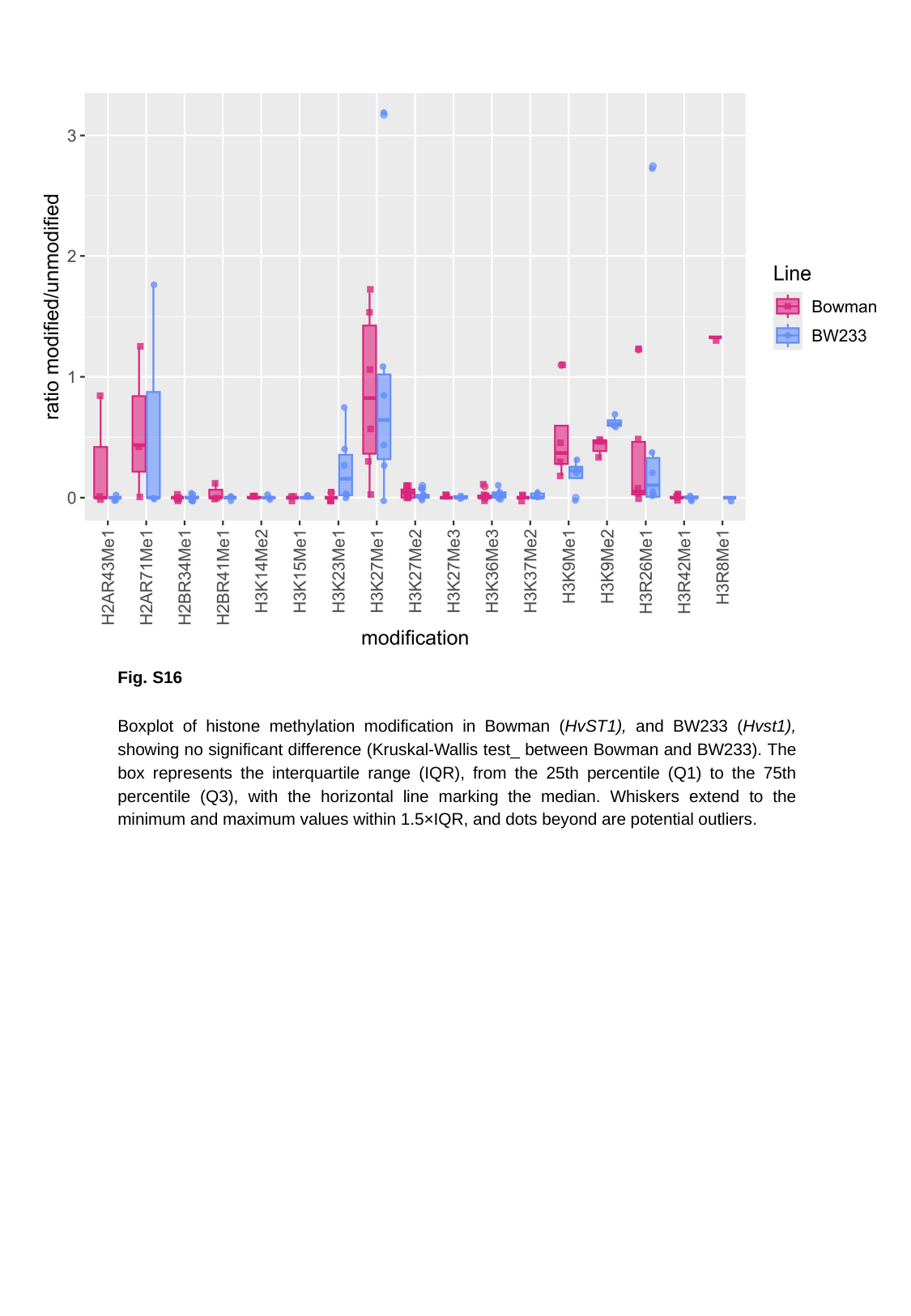

Fig. S16
Boxplot of histone methylation modification in Bowman (HvST1), and BW233 (Hvst1), showing no significant difference (Kruskal-Wallis test_ between Bowman and BW233). The box represents the interquartile range (IQR), from the 25th percentile (Q1) to the 75th percentile (Q3), with the horizontal line marking the median. Whiskers extend to the minimum and maximum values within 1.5×IQR, and dots beyond are potential outliers.

## Slide 18
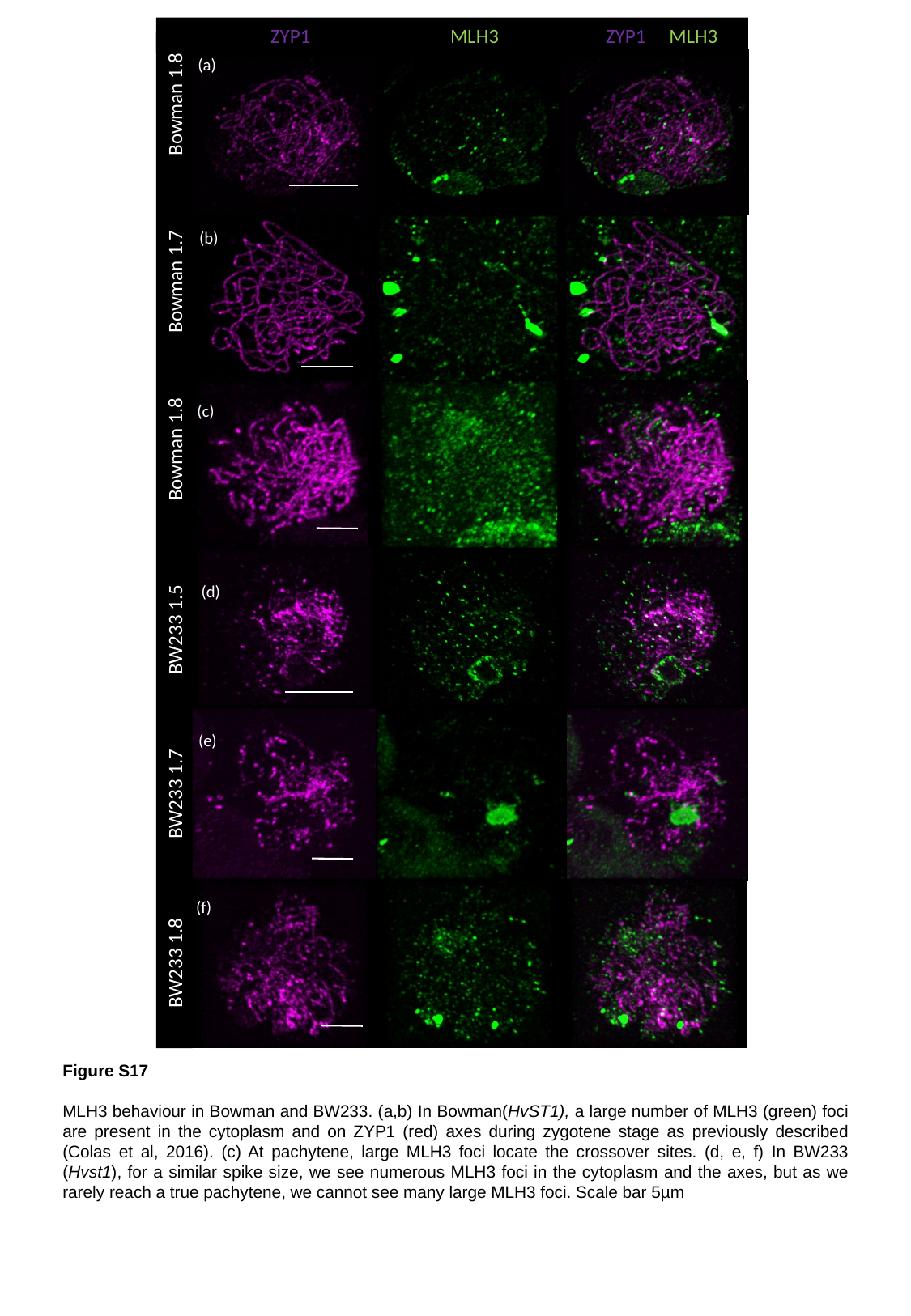

ZYP1 MLH3 ZYP1 MLH3
(a)
(b)
(c)
 BW233 1.8 BW233 1.7 BW233 1.5 Bowman 1.8 Bowman 1.7 Bowman 1.8
(d)
(e)
(f)
( )
( )
( )
( )
( )
( )
( )
( )
( )
( )
( )
( )
Figure S17
MLH3 behaviour in Bowman and BW233. (a,b) In Bowman(HvST1), a large number of MLH3 (green) foci are present in the cytoplasm and on ZYP1 (red) axes during zygotene stage as previously described (Colas et al, 2016). (c) At pachytene, large MLH3 foci locate the crossover sites. (d, e, f) In BW233 (Hvst1), for a similar spike size, we see numerous MLH3 foci in the cytoplasm and the axes, but as we rarely reach a true pachytene, we cannot see many large MLH3 foci. Scale bar 5µm

## Slide 19
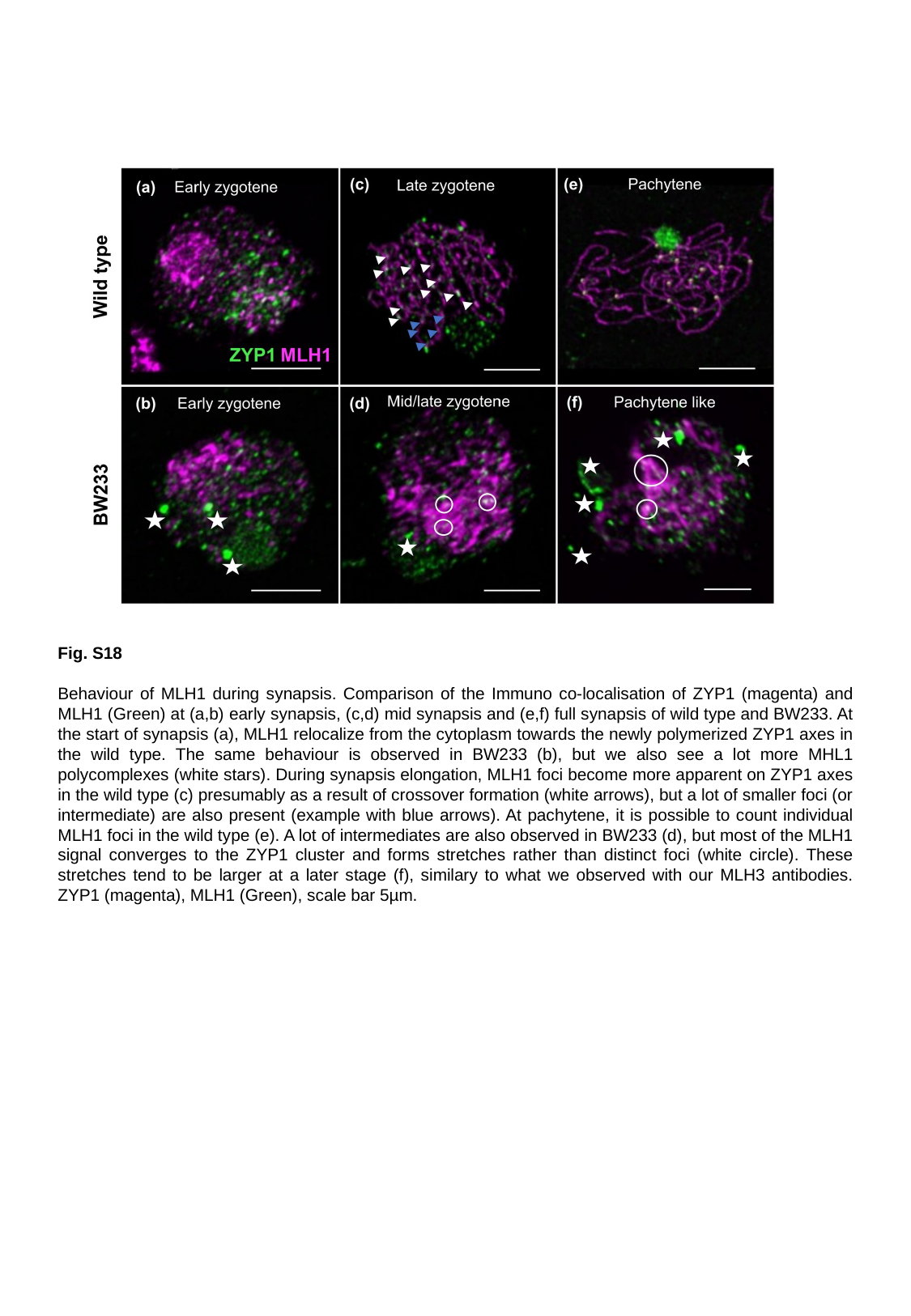

Fig. S18
Behaviour of MLH1 during synapsis. Comparison of the Immuno co-localisation of ZYP1 (magenta) and MLH1 (Green) at (a,b) early synapsis, (c,d) mid synapsis and (e,f) full synapsis of wild type and BW233. At the start of synapsis (a), MLH1 relocalize from the cytoplasm towards the newly polymerized ZYP1 axes in the wild type. The same behaviour is observed in BW233 (b), but we also see a lot more MHL1 polycomplexes (white stars). During synapsis elongation, MLH1 foci become more apparent on ZYP1 axes in the wild type (c) presumably as a result of crossover formation (white arrows), but a lot of smaller foci (or intermediate) are also present (example with blue arrows). At pachytene, it is possible to count individual MLH1 foci in the wild type (e). A lot of intermediates are also observed in BW233 (d), but most of the MLH1 signal converges to the ZYP1 cluster and forms stretches rather than distinct foci (white circle). These stretches tend to be larger at a later stage (f), similary to what we observed with our MLH3 antibodies. ZYP1 (magenta), MLH1 (Green), scale bar 5µm.

## Slide 20
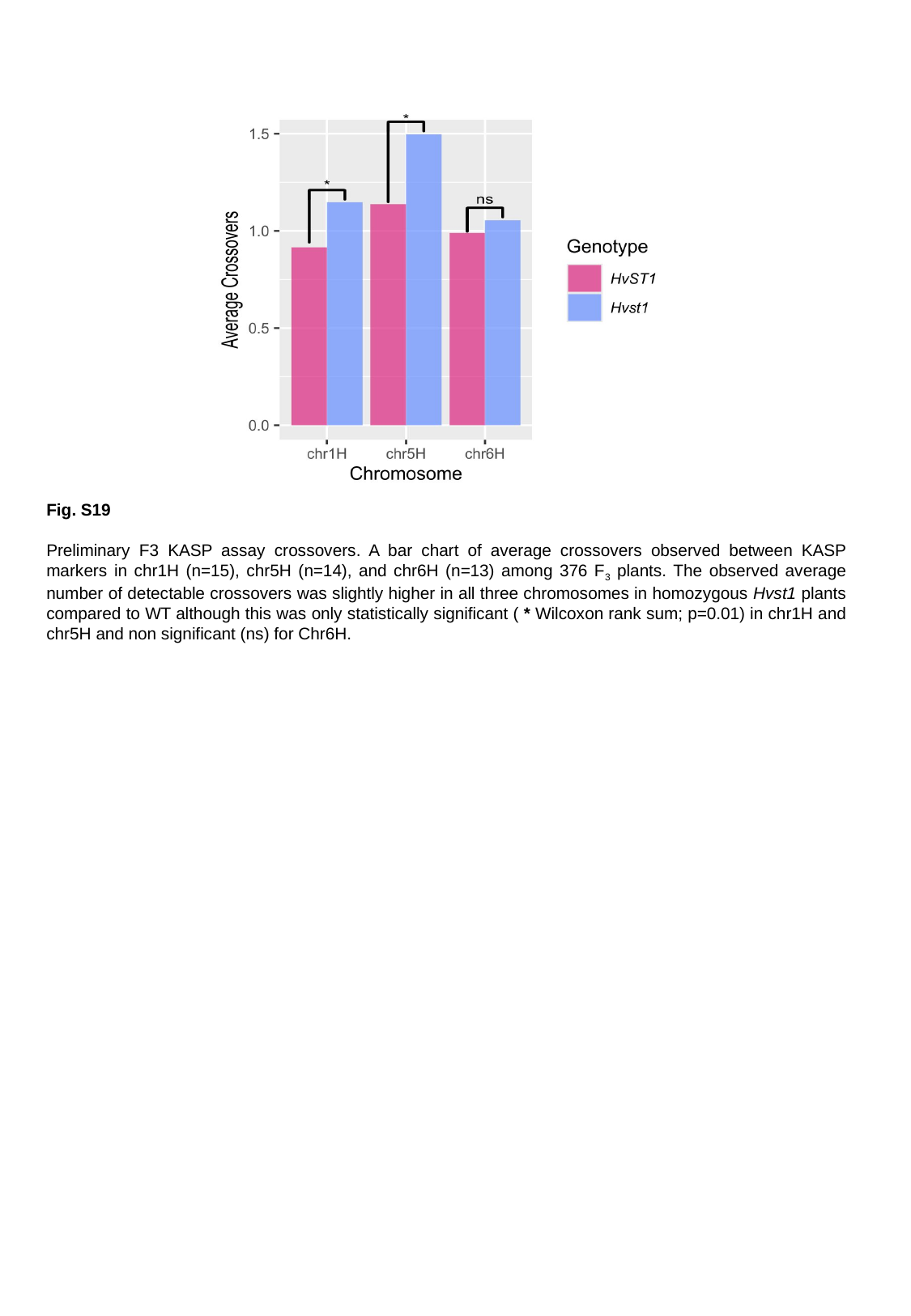

Fig. S19
Preliminary F3 KASP assay crossovers. A bar chart of average crossovers observed between KASP markers in chr1H (n=15), chr5H (n=14), and chr6H (n=13) among 376 F3 plants. The observed average number of detectable crossovers was slightly higher in all three chromosomes in homozygous Hvst1 plants compared to WT although this was only statistically significant ( * Wilcoxon rank sum; p=0.01) in chr1H and chr5H and non significant (ns) for Chr6H.

## Slide 21
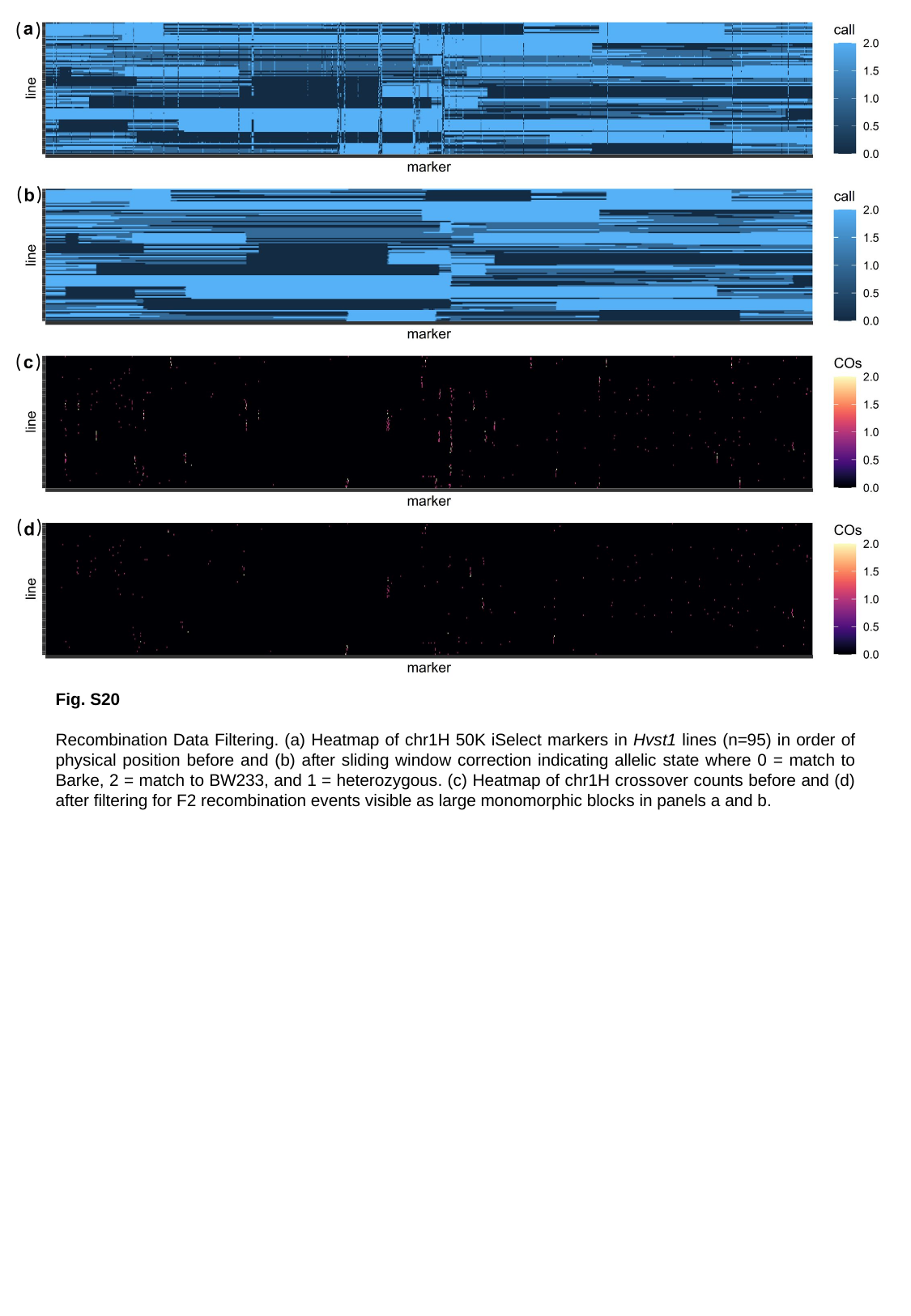

( )
( )
( )
( )
Fig. S20
Recombination Data Filtering. (a) Heatmap of chr1H 50K iSelect markers in Hvst1 lines (n=95) in order of physical position before and (b) after sliding window correction indicating allelic state where 0 = match to Barke, 2 = match to BW233, and 1 = heterozygous. (c) Heatmap of chr1H crossover counts before and (d) after filtering for F2 recombination events visible as large monomorphic blocks in panels a and b.

## Slide 22
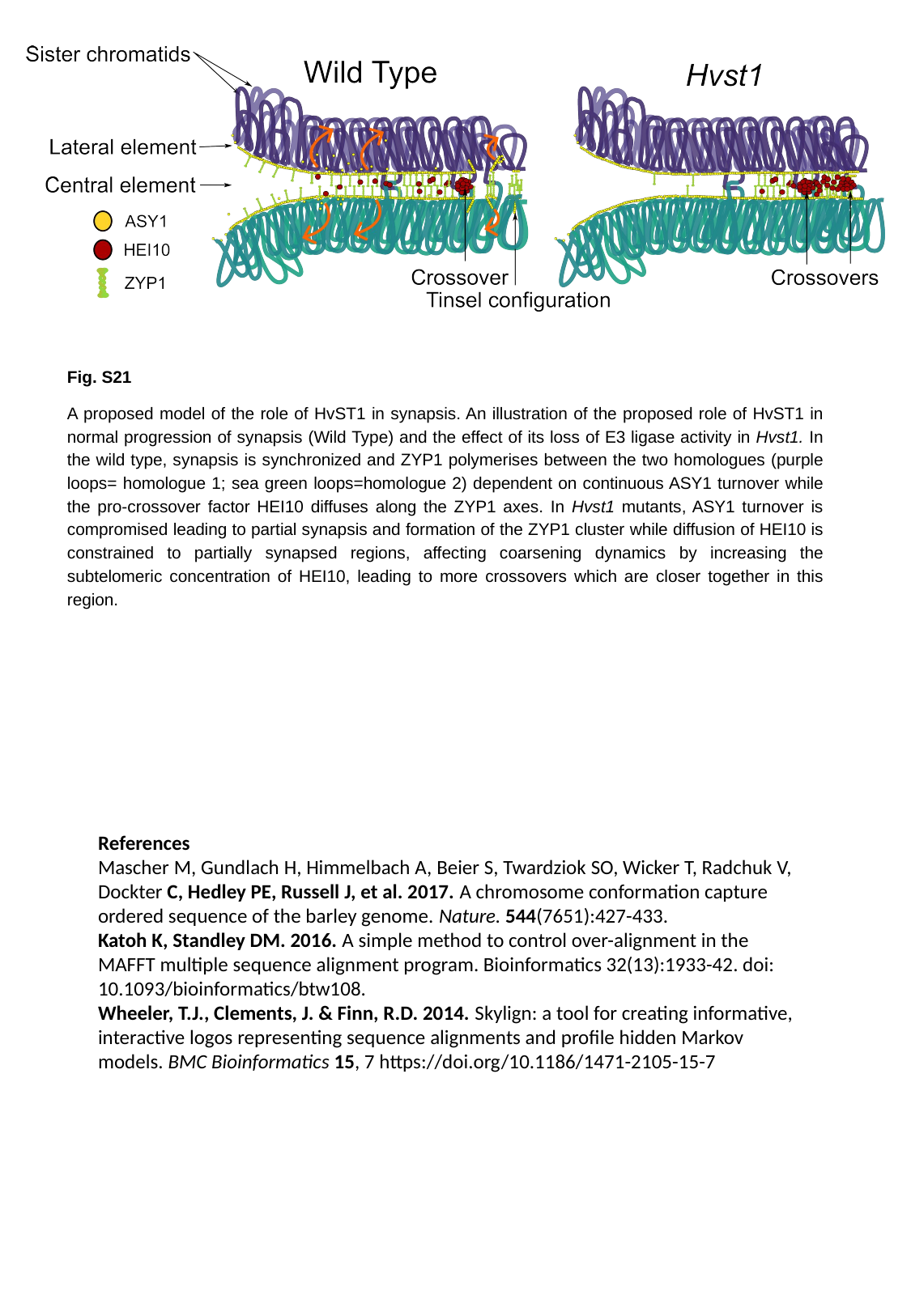

Fig. S21
A proposed model of the role of HvST1 in synapsis. An illustration of the proposed role of HvST1 in normal progression of synapsis (Wild Type) and the effect of its loss of E3 ligase activity in Hvst1. In the wild type, synapsis is synchronized and ZYP1 polymerises between the two homologues (purple loops= homologue 1; sea green loops=homologue 2) dependent on continuous ASY1 turnover while the pro-crossover factor HEI10 diffuses along the ZYP1 axes. In Hvst1 mutants, ASY1 turnover is compromised leading to partial synapsis and formation of the ZYP1 cluster while diffusion of HEI10 is constrained to partially synapsed regions, affecting coarsening dynamics by increasing the subtelomeric concentration of HEI10, leading to more crossovers which are closer together in this region.
References
Mascher M, Gundlach H, Himmelbach A, Beier S, Twardziok SO, Wicker T, Radchuk V, Dockter C, Hedley PE, Russell J, et al. 2017. A chromosome conformation capture ordered sequence of the barley genome. Nature. 544(7651):427-433.
Katoh K, Standley DM. 2016. A simple method to control over-alignment in the MAFFT multiple sequence alignment program. Bioinformatics 32(13):1933-42. doi: 10.1093/bioinformatics/btw108.
Wheeler, T.J., Clements, J. & Finn, R.D. 2014. Skylign: a tool for creating informative, interactive logos representing sequence alignments and profile hidden Markov models. BMC Bioinformatics 15, 7 https://doi.org/10.1186/1471-2105-15-7
