# Supplementary material for: Loss of E3 ligase HvST1 function substantially increases distal crossover frequency
Source: New Phytol. 2025 Nov 30;249(3):1325–41. doi: 10.1111/nph.70757 (PMC12780313; doi:10.1111/nph.70757)
Supplement: Supplementary file 2 — Table S1 KASP primers used to refine the des12.w interval. Table S2 Resequencing primers used to identify polymorphisms for marker development. Table S3 Markers used in preliminary KASP recombination analysis. Table S4 Primers for cloning MLOC_4107/HORVU7Hr1G092570 cDNA. Table S5 Gateway® primers for cloning MLOC_4107/HORVU7Hr1G092570 coding sequence into Escherichia coli expression vector pDEST‐HisMBP. Table S6 CRISPR/Cas9 synthetic guide RNA (sgRNA) oligonucleotides targeting MLOC_4107/HORVU7Hr1G092570. Table S7 Primers for genotyping CRISPR/Cas9 knockout plants. Table S8 Histone methylation summary. Table S9 Change in total crossovers observed per chromosome in WT and BW233 in 50K iSelect SNP chip data. Please note: Wiley is not responsible for the content or functionality of any Supporting Information supplied by the authors. Any queries (other than missing material) should be directed to the New Phytologist Central Office. [file NPH-249-1325-s002.pdf]

**New Phytologist Supporting Information (Table)**

**Article title:**

Loss of E3 ligase *HvST1* function substantially increases distal crossover frequency

**Authors:**
Jamie Neil Orr[1*], Sybille Ursula Mittmann[1,2*], Luke Ramsay[1], Dominika Lewandowska[1], Abdellah Barakate[1], Malcolm Macaulay[1], Nicola McCallum[1], Robbie Waugh[1,2] and Isabelle Colas[1]. *Authors contributed equally to this work*

**Article acceptance date:** 14 October 2025

**Table S1: KASP primers used to refine the des12.w interval**

| iSelect Marker | MLOC | Morex V1 ID | ID | Sequence |
|---|---|---|---|---|
| BOPA1_2462-971 | MLOC_61688 | HORVU7Hr1G088080 | BOPA1_2462-971 Allele-specific primer 1 | GAAGGTGACCAAGTTCATGCTGTAGAGAAATTCCAGACACTGCGT |
| | | | BOPA1_2462-971 Allele-specific primer 2 | GAAGGTCGGAGTCAACGGATTAGAGAAATTCCAGACACTGCGC |
| | | | BOPA1_2462-971 Common, Reverse primer | AACCATGTCATCGAAGTATTTCTGCTCTT |
| SCRI_RS_104566 | MLOC_12475 | HORVU7Hr1G091600 | SCRI_RS_104566 Allele-specific primer 1 | GAAGGTGACCAAGTTCATGCTCAGAACGCCGCTACCCTCTGT |
| | | | SCRI_RS_104566 Allele-specific primer 2 | GAAGGTCGGAGTCAACGGATTGAACGCCGCTACCCTCTGC |
| | | | SCRI_RS_104566 Common, Reverse primer | TTCCTGTGGGGGAGGTTCGGAT |
| SCRI_RS_194841 | MLOC_26843 | HORVU7Hr1G092710 | SCRI_RS_194841 Allele-specific primer 1 | GAAGGTGACCAAGTTCATGCTGCGTGCATGTGATGTGAGCGT |
| | | | SCRI_RS_194841 Allele-specific primer 2 | GAAGGTCGGAGTCAACGGATTGCGTGCATGTGATGTGAGCGC |
| | | | SCRI_RS_194841 Common, Reverse primer | CTTCATTCGCTTTCACAGATCTGAACAA |
| SCRI_RS_127791 | MLOC_67621 | HORVU7Hr1G092810 | SCRI_RS_127791 Allele-specific primer 1 | GAAGGTGACCAAGTTCATGCTAAGGATAAGACGGTCCAACTTGCAA |
| | | | SCRI_RS_127791 Allele-specific primer 2 | GAAGGTCGGAGTCAACGGATTGGATAAGACGGTCCAACTTGCAC |
| | | | SCRI_RS_127791 Common, Reverse primer | TTCCCTTCCCTAAAGTTGCATTAGCAAA |
| BOPA1_1800-1101 | MLOC_18499 | HORVU7Hr1G098440 | BOPA1_1800-1101 Allele-specific primer 1 | GAAGGTGACCAAGTTCATGCTGTACTACATTTACATCATCCGACAGA |
| | | | BOPA1_1800-1101 Allele-specific primer 2 | GAAGGTCGGAGTCAACGGATTGTACTACATTTACATCATCCGACAGT |
| | | | BOPA1_1800-1101 Common, Reverse primer | CTTTCGGTCTWCGAGAACCAAGGAA |

**Table S2: Resequencing primers used to identify polymorphisms for marker development**

| MLOC | Morex V1 ID | ID | Primer | Product | Target |
|---|---|---|---|---|---|
| MLOC_38602 | HORVU7Hr1G092320 | Contig_56831_L08 | TGCTCCTTCGGATATCACTCC | 514 | Exon 4 |
| | | Contig_56831_R08 | TGATGAATAAGACGGACGGG | | |
| MLOC_62816 | HORVU7Hr1G092460 | MLOC_62816_0491C07_L2013 | TCATCCCATGCTCGCTTAC | 819 | SSR |
| | | MLOC_62816_0491C07_R2013 | GAAACGAATAGCATGCAGGA | | |

SSR: Singe Sequence Repeat

**Table S3: Markers used in preliminary KASP recombination analysis**

| Marker | Chromosome | Position cM |
|---|---|---|
| SCRI_RS_155555 | 5H | 2.96 |
| SCRI_RS_192396 | 5H | 13.05 |
| SCRI_RS_190770 | 5H | 30.35 |
| SCRI_RS_8076 | 5H | 48.52 |
| 11_20062 | 5H | 58.27 |
| 11_11355 | 5H | 76.83 |
| SCRI_RS_204275 | 5H | 93.04 |
| SCRI_RS_156633 | 5H | 109.19 |
| SCRI_RS_234720 | 5H | 130.31 |
| SCRI_RS_229673 | 5H | 146.53 |
| SCRI_RS_153238 | 5H | 160.18 |
| 11_20686 | 5H | 176.89 |
| SCRI_RS_4753 | 5H | 194.74 |
| SCRI_RS_217776 | 5H | 197.62 |
| 11_20232 | 6H | 0.63 |
| 11_20262 | 6H | 8.75 |
| SCRI_RS_106920 | 6H | 20.6 |
| 11_10023 | 6H | 24.54 |
| 11_10427 | 6H | 40.17 |
| SCRI_RS_141429 | 6H | 52.68 |
| SCRI_RS_222319 | 6H | 60.5 |
| SCRI_RS_124856 | 6H | 72.52 |
| SCRI_RS_181031 | 6H | 79.42 |
| SCRI_RS_182637 | 6H | 94.81 |
| 12_10704 | 6H | 105 |
| SCRI_RS_169022 | 6H | 116.75 |
| SCRI_RS_124549 | 6H | 124.48 |
| 12_30969 | 1H | 0 |
| SCRI_RS_194326 | 1H | 2.18 |
| 12_30918 | 1H | 13.91 |

| | | |
|---|---|---|
| SCRI_RS_128285 | 1H | 26.53 |
| 11_10744 | 1H | 37.71 |
| SCRI_RS_184784 | 1H | 48.7 |
| 12_30821 | 1H | 60.76 |
| SCRI_RS_145336 | 1H | 73.68 |
| 11_10522 | 1H | 95.76 |
| 11_20625 | 1H | 107.28 |
| SCRI_RS_91596 | 1H | 114.17 |
| 12_11443 | 1H | 127.08 |
| SCRI_RS_163142 | 1H | 137.06 |
| SCRI_RS_199945 | 1H | 141.09 |
| morex_contig_40051_4391_1HS | 1HS | telomeric |
| morex_contig_5842_2000_1HL | 1HL | telomeric |
| morex_contig_229835_15_5HL | 5HL | telomeric |
| morex_contig_37652_453_5HS | 5HS | telomeric |
| morex_contig_41421_6420_6HS | 6HS | telomeric |
| morex_contig_136327_1229_6HL | 6HL | telomeric |
| morex_contig_1569252_2540_5HS | 5HS | telomeric |

cM: Centi Morgan; L: Long arm; S: Short arm

## Table S4: Primers for cloning MLOC_4107/HORVU7Hr1G092570 cDNA

| cDNA primers | Sequence | Product size (bp) |
|---|---|---|
| Nested Pair_ Hvst1 _P1_F (Forward) | CCCTCCTCTGCCGCC**ATG**[*] | 993 |
| Nested_Pair_ Hvst1 _P1_R (Reverse) | CCACCAAGAAACCCCTGC**TCA**[*] | |
| Hvst1 _P2_F (Forward) | CCCCTCCCTCCCGTTCCC | 1061 |
| Hvst1 _P2_R (Reverse) | GAATTGCAACCAAGAAATGCC | |

* The start and stop codons are in bold. bp: base Pair

**Table S5: Gateway® primers for cloning MLOC_4107/HORVU7Hr1G092570 coding sequence into E. coli expression vector pDEST-HisMBP.**

| Primer | Sequence |
|---|---|
| attB1-TEV | GGGGACAAGTTTGTACAAAAAAGCAGGCTCG**GAGAACCTGTACTTTCAG** |
| TEV-MLOC_4107 | **GAGAACCTGTACTTTCAG**GGTatgGCGGGGCTCGCCGAC |
| attB2-MLOC_4107 | GGGGACCACTTTGTACAAGAAAGCTGGGttattaCATCTGCATAGGTGTACCCAACTG |
| attB2-des12 | GGGGACCACTTTGTACAAGAAAGCTGGGttatcaTAACGGCATACCGGGCATGAGTTG |

Gateway sites are underlined, the TEV protease cleavage site is in bold and the start and stop codons are in lower case.

**Table S6: CRISPR/Cas9 synthetic guide RNA (sgRNA) oligonucleotides targeting MLOC_4107/HORVU7Hr1G092570.**

| Primer | Sequence |
|---|---|
| sgRNA_MLOC_4107_L01 | tgttGTTCTTCCTCGGCATCGACG |
| sgRNA_MLOC_4107_R01 | aaacCGTCGATGCCGAGGAAGAAC |
| sgRNA_MLOC_4107_L02 | tgttGACGACATATTCCGGAGCGT |
| sgRNA_MLOC_4107_R02 | aaacACGCTCCGGAATATGTCGTC |

**Table S7: Primers for genotyping CRISPR/Cas9 knockout plants.**

| Primer | Sequence | Product size (bp) | Position |
|---|---|---|---|
| MLOC_4107_CRISPR01_L01 | TACTGCCTCGTATGTTGTGC | 824 | 31909 |
| MLOC_4107_CRISPR01_R01 | GCGATCTCCTCCCACTCG | | 32730 |
| MLOC_4107_CRISPR02_L05 | CCCCTTCTCCTTCGACTCC | 837 | 32544 |
| MLOC_4107_CRISPR02_R05 | AAATGCCACCAAGAAACCCC | | 33380 |
| Cas9-F2 | CCTGTTCGGCAACCTTATCG | 873 | |
| Cas9-R2 | ACCTTCGTCAGCTCGTTGTA | | |

bp: base Pair

**Table S8: Histone Methylation summary**

| | Root (B1WT 2nd) | Pre-meiotic | Early meiosis | Late meiosis |
|---|---|---|---|---|
| Total proteins | 2522 | 1853 | 2185 | 2786 |
| Identified with 2 or more peptides | 1724 | 1178 | 1504 | 1921 |
| Total peptides | 8152 | 5633 | 7495 | 9857 |
| Protein coverage in % | 11.8 | 9.1 | 11 | 12 |
| Histones identified | all* | all* | all* | all* |
| ASY1 identified | N | N | Y | Y |
| Individual methylation events | 8 | 18 | 17 | 19 |
| Methylated peptides | 448 | 888 | 1196 | 1028 |
| Meth peptides ST1/WT | 262/186 | 486/402 | 598/598 | 587/441 |
| Unique methylated proteins | 123 | 211 | 180 | 285 |
| Histones methylated | H3, H2A | H2A, H2B, H3 | H2A, H2B, H3 | H2A, H2B, H3, H4 |

Meth: Methylated; H: Histone; N: No; Y: Yes; *All Histones type after methylation call

**Table S9: change in total crossovers observed per chromosome in WT and BW233 in 50K iSelect SNP chip data**

| Chromosome | *Hvst1* crossovers (n=94) | HvST1 crossovers (n=95) | percent increase *Hvst1* |
|---|---|---|---|
| chr1H | 245 | 130 | 90% |
| chr2H | 324 | 189 | 73% |
| chr3H | 291 | 229 | 28% |
| chr4H | 233 | 131 | 80% |
| chr5H | 312 | 245 | 29% |
| chr6H | 187 | 116 | 63% |
| chr7H | 314 | 127 | 150% |

n: total number; Chr: Chromosome, H: Hordeum
